# Supplementary material for: Unlocking the mysterious polytypic features within vaterite CaCO3
Source: Nat Commun. 2023 Nov 29;14:7858. doi: 10.1038/s41467-023-43625-0 (PMC10687017; doi:10.1038/s41467-023-43625-0)
Supplement: Supplementary file 1 — Supplementary Information [file 41467_2023_43625_MOESM1_ESM.docx]

Supplementary Information

**Unlocking the mysterious polytypic features within vaterite CaCO_3_**

Xingyuan San et al.

^*^Corresponding authors. Email: [xbhu@northwestern.edu](mailto:xbhu@northwestern.edu) (X.H.); [haiyang.niu@nwpu.edu.cn](mailto:haiyang.niu@nwpu.edu.cn) (H.N.); [v-dravid@northwestern.edu](mailto:v-dravid@northwestern.edu) (V.P.D.)

**This PDF file includes:**

Supplementary Text (Note I to IX)

Supplementary Fig. 1 to S25

Supplementary Table 1 to 2

Supplementary References (1 to 23)

**Supplementary Notes**

**Note I. Controversy on structure of vaterite**

Regarding the structure of vaterite, there are many controversies in the literature (Supplementary Table 1). Approximately one hundred years ago, researchers found that the structure of vaterite was distinct from that of calcite and aragonite based on *X*-ray diffraction (XRD) analyses^1^. Then, the cell with hexagonal symmetry (*a=4.11 Å* and *c=8.513 Å*) was derived in 1925 by Olshausen^2^. Based on further XRD analysis, Meyer provided the first crystallographic description*^3^* and proposed an orthorhombic cell model with the space group of *Pnma* and lattice parameters of *a=4.13 Å*, *b=7.15 Å, c=8.48 Å*. Several years later, Kamhi revealed that the vaterite structure has a hexagonal symmetry with space group *P6_3_/mmc* and lattice parameters of *a=4.13 Å*, *c=8.49 Å* using the same XRD technique^4^. Within Kamhi’s hexagonal cell, there is only one unique carbonate group inside the cell, and each atom of the CO_3_^2-^ ion is disordered with a partial occupancy of 1/3, which accounts for considerable disorder. In 1969, Meyer provided a superstructure based on Kamhi’s structure, which has the same space group but different carbonate site symmetry^5^. He believed that the possible stacking disorder of single layers of trigonal symmetry was responsible for diffuse streaks and satellite reflections in the diffraction pattern. Since then, researchers have gradually realized that conventional lab XRD techniques or even advanced synchrotron beam-based diffraction techniques are very limited in solving this structure. Recently, researchers have attempted to address this structural mystery by means of other advanced complementary methods such as theoretical calculations and diffraction tomography, which have resulted in a number of new structure models based on a micro-twinning hypothesis (*Ama2*)^6^, DFT calculations (*P3_2_21*)^7^, automated diffraction tomography (*C2/c* and *P*$\bar{1}$)^8^, molecular dynamics simulations (*P6_5_22*)^9^, high-resolution transmission electron microscopy (HRTEM) data of a biogenic vaterite sample (*P6_3_mmc* of Kamhi-cell in 1963 and unknown cell)^10^ and precession electron diffraction tomography (*C12/c1*)^11^. Furthermore, spectroscopic techniques such as nuclear magnetic resonance (NMR) and Raman spectroscopy are also used to verify the crystal structure of vaterite^12-15^. NMR spectra show that the *P3_2_21* (or *P3_1_21*) model and the monoclinic *C2* model provide the best simultaneous agreement between the experimental and calculated NMR data^12^. The Raman spectrum shows that there are at least three structurally independent carbonate groups in the unit cell of vaterite^13^. More complicated, researchers gradually accepted that vaterite cannot be described based on a single lattice and that there are likely plenty of planar faults including micro-twining and stacking disorder^6,11,16^.

**Supplementary Table 1** Structure information of vaterite CaCO_3_

| Space Group | Lattice Parameters | Methods |
| --- | --- | --- |
| *Pbnm*, *Pnma* | a=4.13 Å, b=7.15 Å, c=8.48 Å α=β=γ=90° | XRD^3^, theory^17^ |
| *P*6_3_22 | a=b=7.14 Å, c=8.52 Å  α=β=90°, γ=120° | XRD^18^ |
| *P*6_3_/*mmc* | a=b=4.13 Å, c=8.49 Å  α=β=90°, γ= 120° | XRD^4,6^ |
| P6_3_/*mmc* | a=b=7.169 Å, c=16.98 Å  α=β= 90°, γ=120° | XRD^5,19^ |
| *P*6_5_22, *P*3_5_21, *P6_5_* | a=b=7.29 Å, c=25.30 Å  α=β= 90°, γ=120° | XRD^20^, theory^7,9,21^ |
| *Ama*2 | a=8.47 Å, b=7.16 Å, c=4.13 Å  α=β=γ=90° | Theory^6^ |
| *P*2_1_2_1_2_1_ | a = 4.37 Å, b = 6.58 Å, c = 8.43 Å  α=β=γ=90° | Theory^7^ |
| *C*2, *Cc*, *C*2/*c* | a=12.17 Å, b=7.12 Å, c = 9.47Å α=γ=90°, β=118.94° | ED^8^, theory^22^ |
| *C*1, *C*$\bar{\boldsymbol{1}}$ | a = 12.36 Å, b = 7.11 Å, c = 25.74 Å  α = 90.43°, β = 99.88°, γ = 90.29° | ED^8^, theory^22^ |

**Note II. Different marking methods for vaterite**

In addition to various unit cells proposed to explain the crystal structure of vaterite, two marking methods were developed to describe both ordered and disordered models with any stacking sequence according to the characteristics of each carbonate layer^22,23^. The planes of carbonates with half of the specific occupation of triangular prisms made up by the calcium atoms are labelled with the letters A, B, and C, while each letter indicates an orientation of carbonates. Another half of the occupation with carbonate planes is labelled with A′, B′ or C′ in line with the different orientations of carbonates. Supplementary Fig. 6a presents the projections of a stacking model onto the *xz*-plane of the six possible permutations of carbonates while odd-number layers and even-number layers are denoted by different colors. The carbonate layers with the same color are labelled with the same type of letters, e.g., (A, B, C) or (A′, B′, C′), which correspond to half of the specific occupation as shown in Supplementary Fig. 6b. However, this marking method has its own drawbacks. By rotating the vaterite along the *z*-axis with an integer multiple of 60°, a structure with calcium atoms and carbonates like the structure before rotation is obtained since the calcium atoms exhibit a pseudohexagonal lattice. However, the letters marked for each layer will not retain their sequences as the structure rotates. In other words, each direction requires a certain set of letters to label the sequence of carbonates. For instance, the labels of the vaterite with the *C2* space group in the three directions are shown in Supplementary Fig. 7a. We find that the corresponding labels of the projections obtained in different directions are not the same, even though they share the same stacking sequence.

In Christy’s work^23^, the relation of orientations between two layers of carbonate separated by one another is used to label the stacking sequence. The marking method is shown in Supplementary Fig. 8. In this way, the two layers of carbonates separated by one another apart from +120°, -120° or 0° can be marked as “+”, “−”, and “0” while the middle layer is indifferent. For instance, the *C2* structure and *P3_2_21* structure can be marked by ‘+−+−+−’ and ‘++++++’, respectively, while reflection in a vertical mirror plane will interconvert + and – operations. This method can describe any stacking sequence well with the projections of different directions. Supplementary Fig. 7b shows the labels of the vaterite with the *C2* space group in three directions, while their labels are identical.

**Note III. Experimental indistinguishability of three basic monoclinic structures**

**Supplementary Table 2.** Extinction rule for the space groups of *C2*, *Cc* and C*2/c*.

|  | (hkl) | (h0l) | (0kl) | (hk0) | (h00) | (0k0) | (00l) |
| --- | --- | --- | --- | --- | --- | --- | --- |
| *C2* | h+k=2n | h=2n | k=2n | h+k =2n | h=2n | k=2n |  |
| *Cc* | h+k=2n | h, l=2n | k=2n | h+k =2n | h=2n | k=2n | l=2n |
| *C2/c* | h+k=2n | h, l=2n | k=2n | h+k =2n | h=2n | k=2n | l=2n |

In Demichelis’s work^22^, a basic monoclinic structure with three possible space groups (No.5, *C2*; No.9, *Cc*; No.15, *C2/c*) have been proposed based on theoretical calculations. Although the proposed basic monoclinic structure has three possible space groups, their lattice parameters are similar to each other with *a*=*12.3* Å, *b*=*7.13* Å, *c*=*9.4* Å, *β*=115.48°. Regarding the space groups of *C2*, *Cc* and *C2/c*, it is impossible to distinguish them based on the electron diffraction patterns (EDPs). The extinction rule of space groups *C2*, *Cc* and *C2/c* in EDPs are listed in Supplementary Table 2, where *n* equals positive integer. The space groups *Cc* and *C2/c* share the same extinction rule. Thus, the diffraction patterns should have the same distributions. That is why we cannot distinguish them based on EDPs. Meanwhile, considering the double diffraction resulting from the dynamic effect, we cannot distinguish *C2* from *Cc* or *C2/c*, although there are some slight differences in their extinction rules. The simulated EDPs of the vaterite structure with the above three space groups along some low index directions are shown in Supplementary Fig. 4 and Fig. 5. It is found that the distribution of diffraction patterns with above three space groups along [010], [103], [001], and [101] are the same. Only along the [100] direction, there are slight differences (See Supplementary Fig. 4a, 4c, 4e). In the simulated EDPs, (00*l*) with *l* equal to an odd number does not exist in Supplementary Fig. 4a-d but occurs in Supplementary Fig. 4e, 4f. However, due to the unavoidable dynamic effects when electron beams interact with the samples, these patterns should always exist. Thus, we cannot distinguish them based on the distributions of the diffraction patterns. Indeed, there may be slight differences in the intensities of the reflection patterns for different space groups. However, it is very challengeable to quantify the intensities of diffractions in conventional electron diffraction since the intensities depend on many factors such as the beam intensity, sample thickness and orientation deviations of local areas. Therefore, it is very challenging to distinguish the above three possible vaterite structures based on only the experimental EDPs.

**Note IV. Inherent relationships between the monoclinic lattice and hexagonal lattice**

The disordered Meyer structure with a hexagonal lattice (*P6_3_/mmc*; *a*=*b*=*7.169* Å, *c*=*16.98* Å) has been extensively discussed^5,19^. Here, based on elaborate crystallographic considerations, we uncover the inherent relationships between the abovementioned monoclinic lattice and Meyer’s hexagonal lattice. Since we cannot distinguish the space groups among *C2*, *Cc*, and *C2/c* experimentally, here we simply labeled them as a monoclinic lattice. As shown in Supplementary Fig. 11, along the [103]_m_ direction, where the subscript *m* represents the monoclinic lattice, the projection of the monoclinic lattice has a pseudohexagonal feature. The lengths of the [010]_m_, [110]_m_ and [1$\bar{1}$0]_m_ directions are approximately the same. Meanwhile, since the Meyer’s hexagonal structure has 6-fold symmetry along the [001]_H_ direction, where the subscript H represents the hexagonal lattice, it is reasonable to deduce that there is similarity between the lattices of Meyer’s hexagonal structure and monoclinic structures. Interestingly, it is found that the lengths of the [010]_m_, [100]_m_, and [206]_m_ directions are almost the same as those of the [010]_H_, [210]_H_, and [003]_H_ directions, as indicated in Supplementary Fig. 11. Thus, the inherent orientation relationships between the hexagonal Meyer lattice and monoclinic lattice can be described using the following matrix transformation.

$$\left( \begin{matrix} u \\ v \\ w \end{matrix} \right)H=\left( \begin{matrix} 2 & 0 & -\frac{2}{3} \\ 1 & 1 & -\frac{1}{3} \\ 0 & 0 & \frac{1}{2} \end{matrix} \right)\left( \begin{matrix} u \\ v \\ w \end{matrix} \right)m$$

**Note V. Possible orientational variants within the monoclinic lattice**

Considering the existence of the pseudohexagonal feature within the monoclinic lattice along the [103]_m_ direction, as shown in Supplementary Fig. 11, it is reasonable to deduce that there may be three possible orientation variants within the monoclinic structure, which is formed due to the 60° (or 120°) rotation along the [103]_m_ zone-axis. As revealed in Supplementary Fig. 11, because of continuous 60° clockwise rotation, the [010]_m_ direction will change to the [110]_m_ direction and then to the [1$\bar{1}$0]_m_ direction. Meanwhile, the [200]_m_ direction will change to the [1$\bar{3}$0]_m_ direction and then to the [$\bar{1}\bar{3}$0]_m_ direction. Thus, the directions within three variants (V1, V2, V3) can be correlated using the following matrix transformation.

$$\left( \begin{matrix} u \\ v \\ w \end{matrix} \right)V2=\left( \begin{matrix} \frac{1}{2} & \frac{1}{2} & \frac{1}{6} \\ -\frac{3}{2} & \frac{1}{2} & \frac{1}{2} \\ 0 & 0 & 1 \end{matrix} \right)\left( \begin{matrix} u \\ v \\ w \end{matrix} \right)V1$$

$$\left( \begin{matrix} u \\ v \\ w \end{matrix} \right)V3=\left( \begin{matrix} \frac{1}{2} & -\frac{1}{2} & \frac{1}{6} \\ \frac{3}{2} & \frac{1}{2} & -\frac{1}{2} \\ 0 & 0 & 1 \end{matrix} \right)\left( \begin{matrix} u \\ v \\ w \end{matrix} \right)V1$$

**Note VI. Index of serial electron diffraction patterns**

Intuitively, all the EDPs shown in Fig. 2a-f have a clear feature that the intensity of the reflections is not uniform. There are clearly 2 sets of patterns in each figure. As indicated in Supplementary Fig. 20a which is a local magnification of Fig. 2a, one set of patterns has a very high intensity (Set-I) indicated by cyan circles, and the other set (Set-II) of patterns has a relatively weak intensity indicated by magenta circles. The intensity of Set-I patterns is >10 times larger than that of the Set-II patterns. Here, we first only consider the relative positions of the reflections but ignore the intensity difference. Simulations of the EDPs of Meyer’s hexagonal structure and the monoclinic structure along some major zone-axes are shown in Supplementary Fig. 12-14. Detailed analysis demonstrates that the EDPs shown in Fig. 2a can be tentatively indexed based on the hexagonal Meyer structure. However, the remaining EDPs in Fig. 2 can only be indexed based on the monoclinic structure. The reason why Fig. 2a cannot be indexed based on the monoclinic structure is the existence of Set-II weak patterns. For example, Set-I patterns such as {030}_H_, {300}_H_ and {330}_H_ in Fig. 2a can be indexed as the [103]_m_ zone-axis based on the monoclinic lattice (See Supplementary Fig. 13a). However, Set-II patterns such as {100}_H_, {200}_H_, {110}_H_ and{220}_H_ should not occur along the [103]_m_ zone-axis.

The relative distributions of the EDPs shown in Fig. 2b, 2d and 2f are the same except for a 60° rotation along the viewing direction. All of them can be indexed based on the monoclinic structure and correspond to the [001]_m_ zone-axis. Considering the experimental tilting angles shown in Supplementary Fig. 2, it is found that the intersection angle between positions B (D) and D (F), which correspond to the EDPs of Fig. 2b (2d) and 2d (2f) respectively, is 60°. The relative distribution of EDPs shown in Fig. 2c and 2e are the same as well, and the intersection angle between position C (corresponding to Fig. 2c) and E (corresponding to Fig. 2e) is 60° as well. Thus, it is deduced that there are likely three orientation variants within the vaterite structure. These three orientation variants are generated due to a 60° rotation along the [103]_m_ zone-axis, which agrees well with the above crystallographic considerations in Note V. The orientation relationship among variants I, II and III can be described using the matrix listed in Note V. When variant I is tilted to the [001]_m_^V1^ direction, variants II and III will be orientated along the [136] _m_^V2^ and [1$\bar{3}$6] _m_^V3^ directions respectively. The simulated EDPs along the [001]_m_^V1^, [136] _m_^V2^ and [1$\bar{3}$6] _m_^V3^ directions are shown in Supplementary Fig. 12a, 12c, and 12e, respectively. By overlapping them, the simulated patterns reproduce the experimental results shown in Fig. 2b, 2d and 2f. Set-I patterns with higher intensity are shared by variants I, II and III. However, Set-II patterns only occur in variant I along the [001]_m_^V1^ direction. Thus, Set-I patterns can have a higher intensity and Set-II patterns have a relatively lower intensity. Similarly, when variant I is tilted along the [101]_m_^V1^ direction, variants II and III will be orientated to the [2$\bar{3}$3] _m_^V2^ and [233] _m_^V3^ directions respectively. Overlapping their simulated EDPs (Supplementary Fig. 12b, 12d, 12f) will reproduce the experimental results shown in Fig. 2c and 2e perfectly.

Now, let us move on to the EDPs shown in Fig. 2a again, which can be tentatively labelled as [001]_H_ for simplicity. Since the hexagonal Meyer structure and monoclinic structure are closely related, the [001]_H_ direction in the hexagonal Meyer structure is equivalent to the [103]_m_ direction in the monoclinic structure (See Supplementary Fig. 11). Since variants I, II, III are formed due to a 60° rotation along the [103]_m_ zone-axis, EDPs along the [103]_m_ direction for variants I-III should be the same (See Supplementary Fig. 13a). Overlapping the [103]_m_ EDPs can only generate the Set-I patterns. However, the occurrence of the Set-II patterns in the hexagonal Meyer structure is due to the disordered arrangement of carbonate (CO_3_^2-^). Along the [103]_m_ direction, after continual 60° rotation, three types of carbonates seem to be arranged in a “disordered” way. Thus, this will introduce additional Set-II patterns, which are confirmed by the simulated EDPs shown in Fig. 20b based on our theoretically grown vaterite with polytypic structural features.

**Note VII. Structural and energy characteristics of carbonates with different stackings**

Here, we will clarify how the stacking orders of layers with different carbonate orientations along the z-axis affect the stability of vaterite. As stated in the main text, the carbonates in one layer have three different possible orientations. If only looking into the stacking of two contiguous layers, any stacking order is equivalent due to rotational symmetry, as shown in Supplementary Fig. 9. Nevertheless, if we consider the stacking orders of two carbonate layers separated by one another, as introduced in Supplementary Fig. 8c, the situation is quite different. As shown in Supplementary Fig. 10b and 10c, when the two layers of carbonates have different orientations, corresponding to the stacking order of “+” or “−”, the carbonates and calcium atoms are arranged like a honeycomb on the projection of the *xy*-plane. On the other hand, as shown in Supplementary Fig. 10d, when the two layers of carbonates have the same orientation, corresponding to the stacking order of “0”, their projections on the *xy*-plane are apparently coincident.

The above discussion has illustrated that the scenario of the stacking order “0” is entirely different from that of “+” or “−”. Such difference can be further elucidated from the perspective of energy. We run MD simulations with the initial configurations corresponding to different scenarios using the DNN model at 300 *K*, and their potential energies as a function of time are shown in Supplementary Fig. 19. One can find that if the stacking sequences only contain “+” and/or “-”, the average energies are almost the same (within 2.5 *meV/f.u.*, less than the error margin of the DNN model). In contrast, the energy of the stacking sequence containing only “0” is approximately 41 *meV/f.u.* higher than the former ones. The result demonstrates that the sequence order “0” is energetically unfavorable.

**Note VIII. Difficulties in characterizing the structure of vaterite with synchrotron X-ray or neutron diffraction techniques**

Synchrotron X-ray or neutron diffraction techniques are very powerful toolsets for solving complex crystal structures. Researchers have also been tried to resolve the structure of vaterite using synchrotron X-ray^8,11,21^ and neutron diffraction neutron diffraction^20^. But the derived conclusions are still controversial. Based on the neutron diffraction data, Chakoumakos *et al.*^20^ indicated that the hexagonal structural model with space group *P6_5_22* provides the best fit, which agrees well with the model provided based on synchrotron X-ray diffraction data^21^. However, based on the synchrotron X-ray data, Mugnaioli *et al.*^8^ proposed that vaterite structure likely could be labeled by a monoclinic structure (*a* = 1.217 nm, *b* = 0.712 nm, *c* = 2.532 nm, *β* = 99.228°) and Steciuk *et al.*^11^ however proposed that vaterite should be labeled by a superspace group.

Previous results demonstrated that synchrotron X-ray and neutron diffraction techniques are very limited here and cannot provide exclusive conclusions for vaterite structure. The main reason is that the structure of vaterite is heavily faulted as shown in Supplementary Fig. 15. We propose that vaterite should be regarded as polytypic structure, in which the stacking is random along [103]_m_ direction. There are not only many stacking disorders but also three orientation domains. Within even 1-2 nm, stacking disorders and/or stacking faults occurs. The orientation domain which was resulted from localized stacking orders is also very small (< 3 nm) as shown in Fig. 4b. The spatial resolution of synchrotron X-ray and neutron diffraction is around 10-30 nm and 20 µm, respectively. Within this scale, there are always lots of stacking disorders in vaterite. Thus, synchrotron X-ray and neutron diffraction techniques can only provide an averaged structural information, where many nanoscale features including stacking disorder and domains are averaging out. In summary, as for vaterite, we believe that the single variant size is too small and the density of stacking faults are too high that it is very challenging to use the X-ray diffraction and neutron diffraction to resolve its structure, particularly the atomic modulations.

We ensured that the resolution of the powder diffractometer used for this experiment was high enough to resolve broadening of reflections due to the defects. Supplementary Fig. 22 shows the experimental powder data of the sample against a Silicon standard (NIST 640d) which contains no strain/defects. Instrumental resolution is narrower than the resolution needed to resolve the broad peaks observed for the sample. We believe the superior resolution from a synchrotron source will not provide additional unique information given that the broadening of the peaks for this particular sample is intrinsically large. Additionally, we performed simulations of powder patterns using the proposed model for X-rays and neutrons for the same wavelength of 1.54187 Å. As shown in Supplementary Fig. 23 and 24, it is apparent that neither X-rays nor neutrons give significant contrast in the entire *q*-range. Certain regions appear better for X-rays and other regions appear better for neutrons.

**Note IX. High-symmetry structure and low-symmetry structure identification and collective variable**

In previous work, Demichelis *et al.^7,22^* proposed several low-symmetry structures (LS) with greater stability based on the density functional theory (DFT) calculations, while the earlier proposed high-symmetry structures (HS) correspond to the transition state. The difference between HS and LS is the tilt angle of the carbonates, which also leads to a slight shift in the positions of calcium atoms.

According to the different tilt angles of carbonates, we therefore define the difference between the coordinates of the two overlapping oxygen atoms of one layer in the *z*-axis direction as *Δz_1_* as shown in Supplementary Fig. 25. Since the tilting feature of carbonates occurs in two layers as a period in the *z*-axis direction, the coordinate difference in the *z*-axis direction of two overlapping oxygen atoms of adjacent layers is defined as *Δz_2_* according to the same definition. We define *Δz_1_-Δz_2_* as the collective variable (CV) to describe the symmetry of our system. This definition has the advantage that when the CV is zero, the structure corresponds to HS. When the CV is not zero, the structure corresponds to LS. However, using only a few carbonates to define the symmetry of the system will inevitably lead to large errors. Therefore, we use this method to redefine the order parameter by using the coordinate information of all overlapping carbonates and oxygen atoms in the system such as

$Q=\frac{2}{mn}\sum_{i=1}^{n} \sum_{k=1}^{\frac{m}{2}} (\Delta z_{2k-1,i}-\Delta z_{2k,i})$,

where *m* is the number of layers of carbonates in the *z*-axis of the system, and *n* is the number of pairs of carbonates with two overlapping oxygen atoms in one layer. In this way, two-thirds of the carbonates in the system are used for the CV calculations.

**Supplementary Figures**


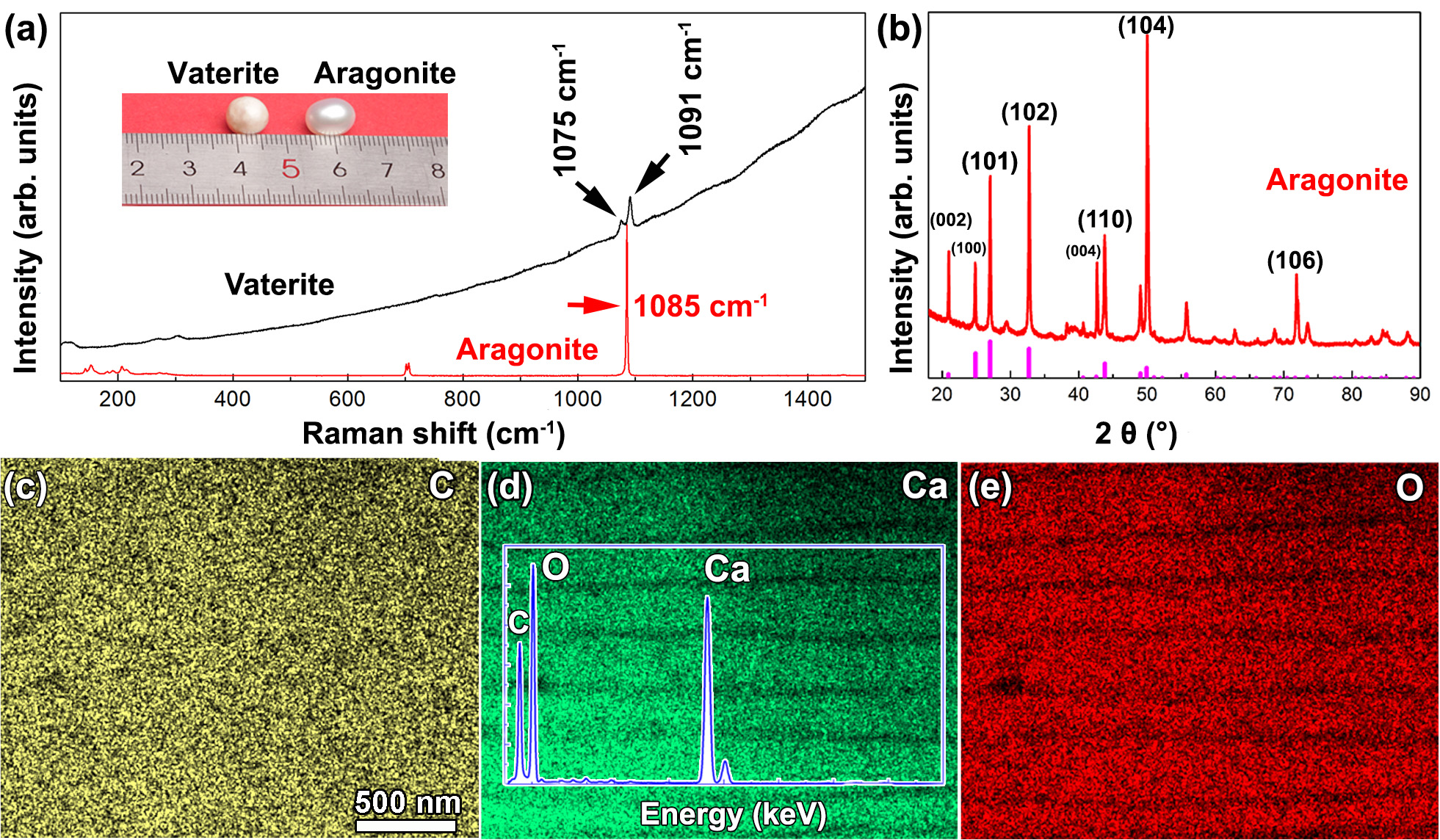


**Supplementary Fig. 1. Microstructure and chemical features of aragonite.** (a) Raman spectra of the pearls with aragonite and vaterite structures. The inset microscopic image shows the general appearance of the lackluster and normal pearls. (b) XRD patterns of the freshwater lackluster pearl. Bottom vertical pink lines show the peak positions of vaterite structure from PDF-33-0628. (c-e) C, Ca and O maps of vaterite, corresponding to the region shown in Fig. 1b. The inset in (d) is the EDS spectrum of vaterite.


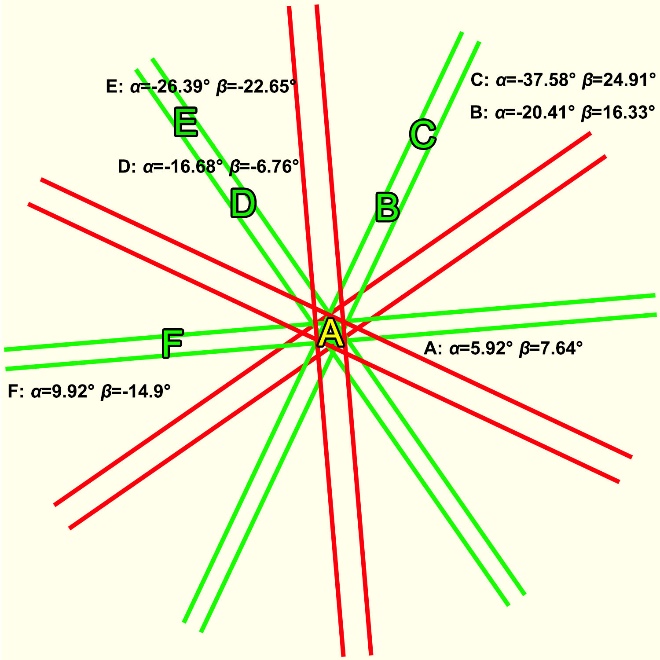


**Supplementary Fig. 2. Original experimental tilting data for the serial EDPs shown in Fig. 2.**


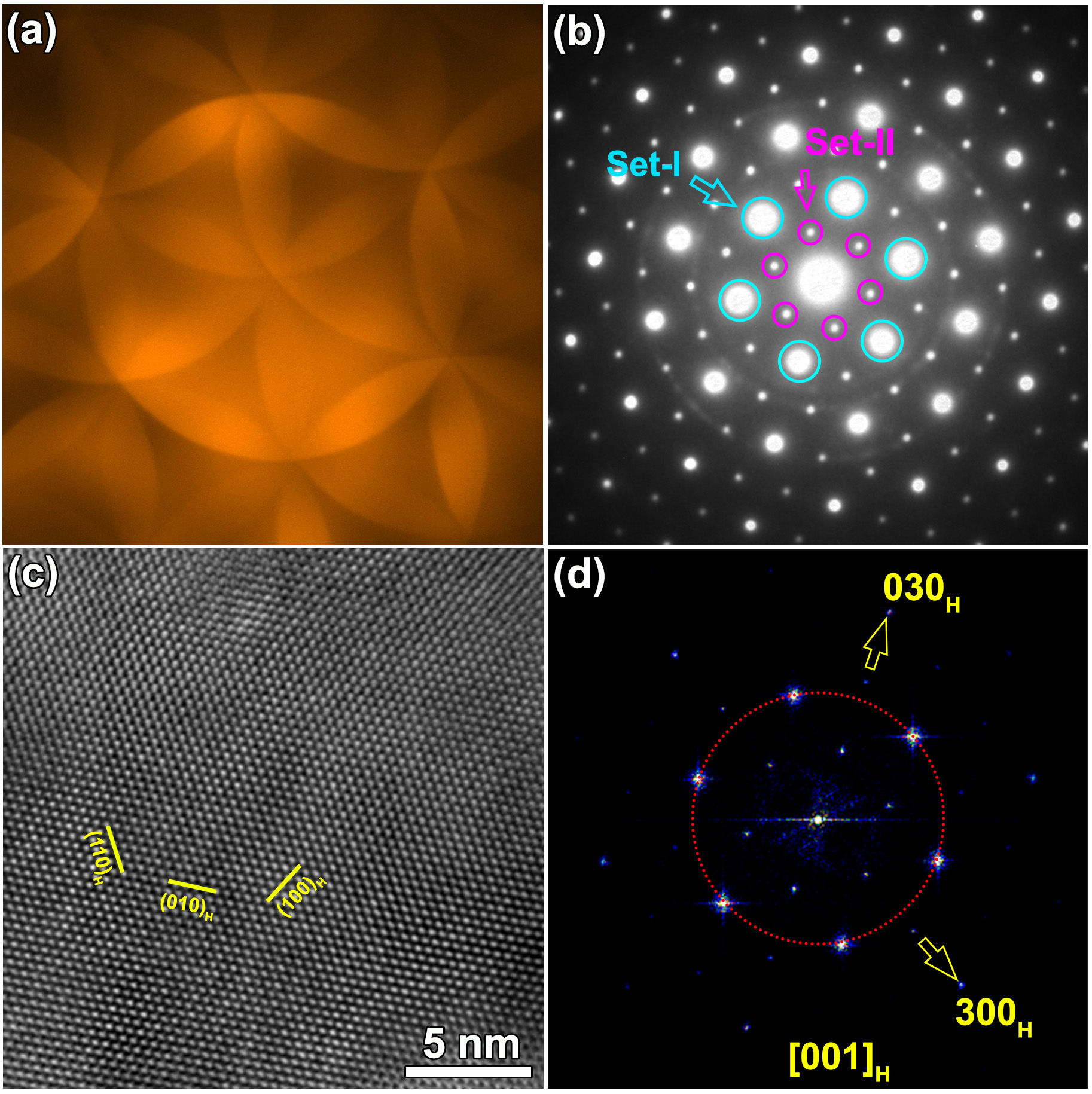


**Supplementary Fig. 3. Pseudo-hexagonal symmetry within vaterite.** (a) Convergent beam electron diffraction patterns of vaterite along the same direction axis as Fig. 2b. (b) Duplicate EDPs of Fig. 2b. Cayan circles indicate some of the Set-I patterns with higher intensity. Magenta circles indicate some of the Set-II patterns with relatively lower intensity. (c) Atomic resolution TEM images of vaterite along the [103]_m_ direction. (d) Digital fast Fourier transform (FFT) patterns of the HRTEM image in (c).


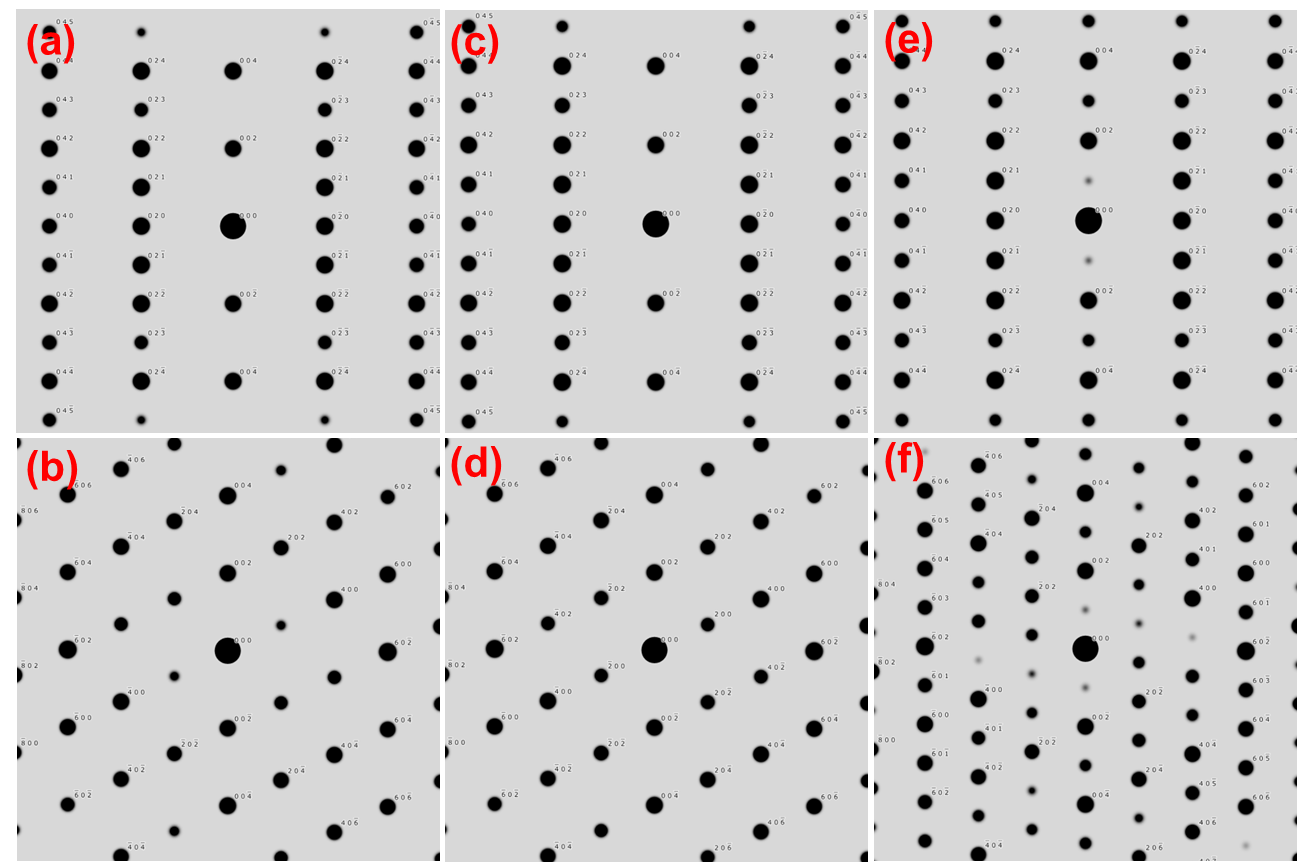


**Supplementary Fig. 4. Comparisons of simulated diffractions for *C2*, *C2/c* and *C2* structure.** Simulated EDPs of the vaterite CaCO_3_ with (a, b) the space group of *C2/c* along the [100], [010] directions; (c, d) the space group of *Cc* along the [100], [010] directions; (e,f) the space group of *C2* along the [100], [010] directions.


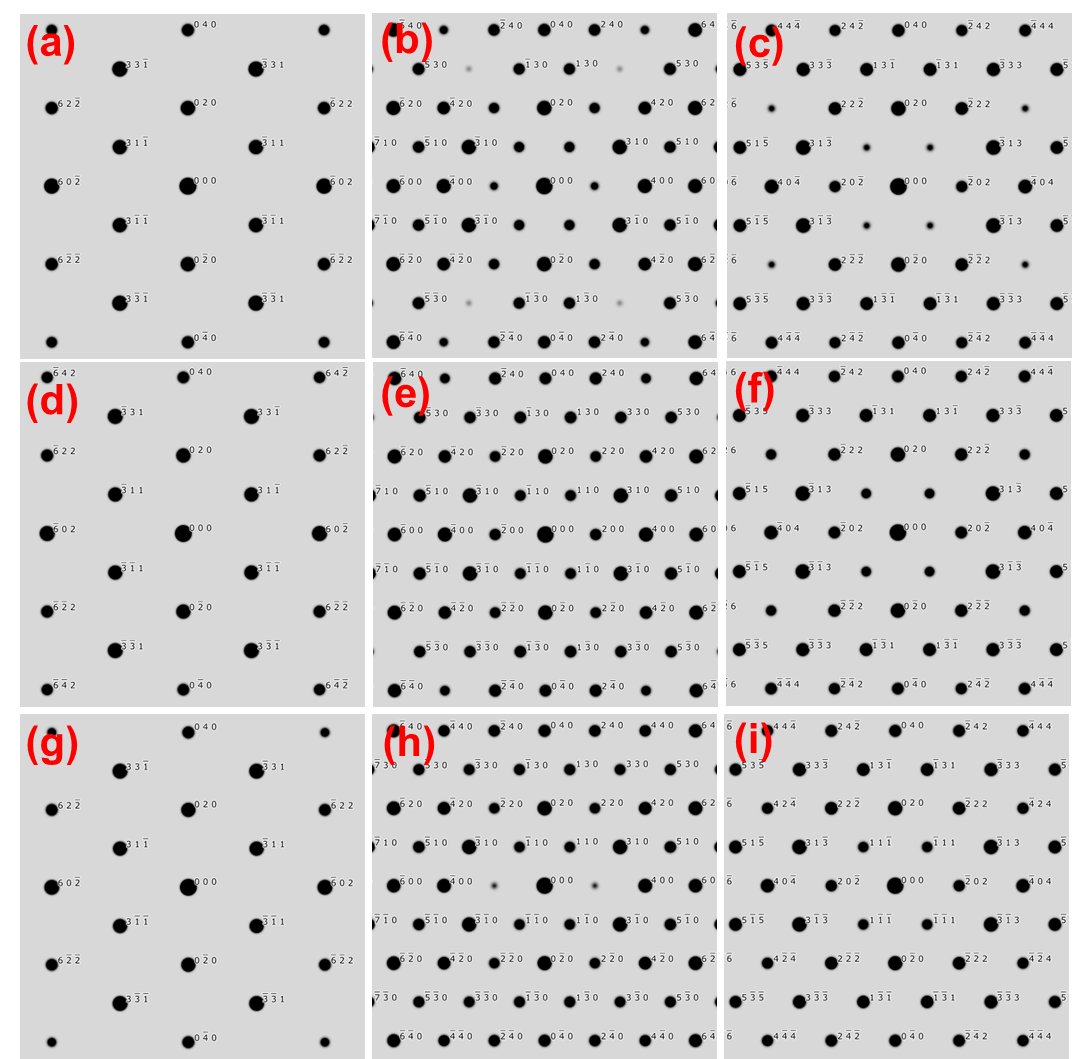


**Supplementary Fig. 5. Comparisons of simulated diffractions for *C2*, *C2/c* and *C2* structure.** Simulated EDPs of the vaterite CaCO_3_ with (a-c) the space group of *C2/c* along the [103], [001] and [101] directions; (d-f) the space group of *Cc* along the [103], [001] and [101] directions; (g-i) the space group of *C2* along the [103], [001] and [101] directions.


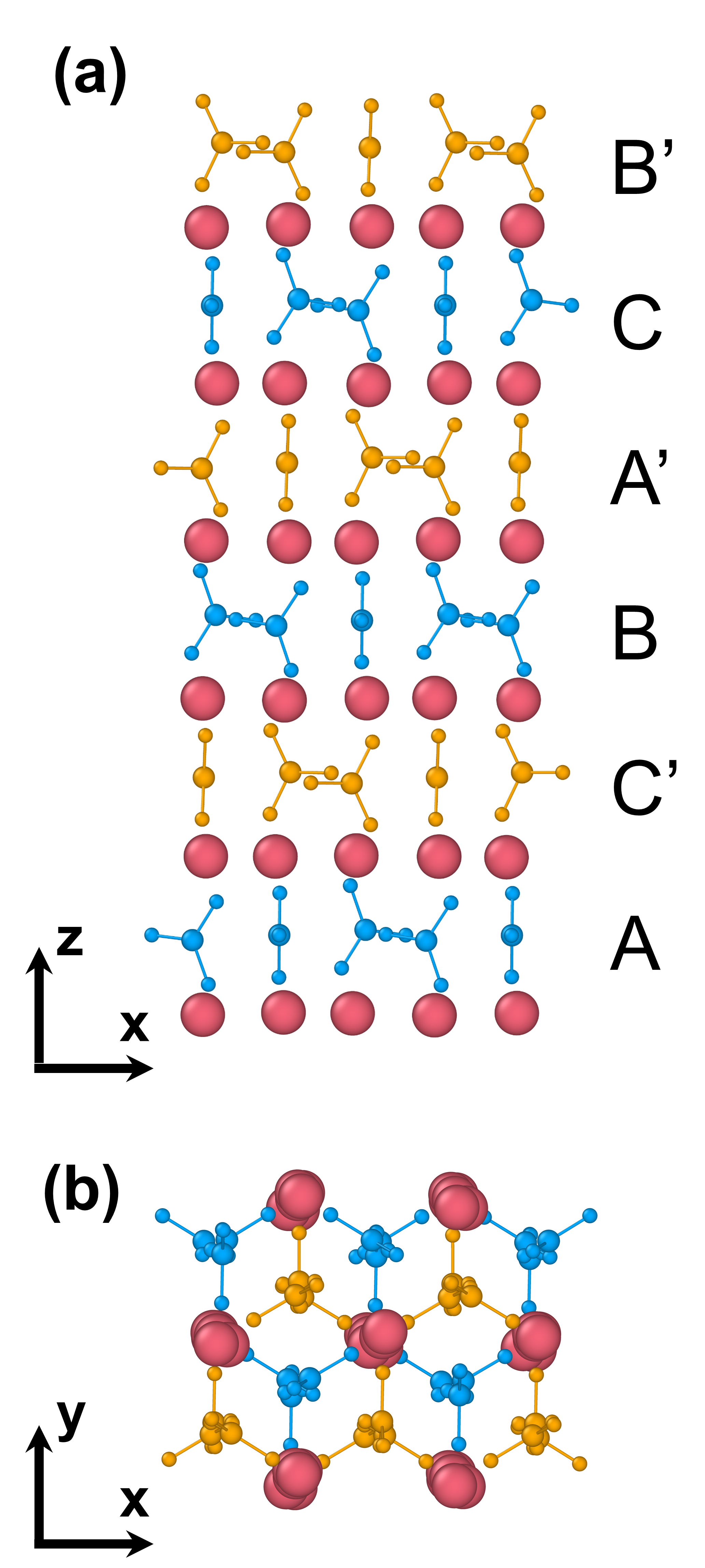


**Supplementary Fig. 6. Structural projections of a stacking model on different planes.** (a) *xz*-plane. (b) *xy*-plane. In this model, all six possible permutations of carbonates are labelled with different letters. The carbonates in the odd layers and even layers are colored blue and brown, respectively. The carbonates with the same color are approximately ±120° or 0° apart from each other in the *xy*-plane.


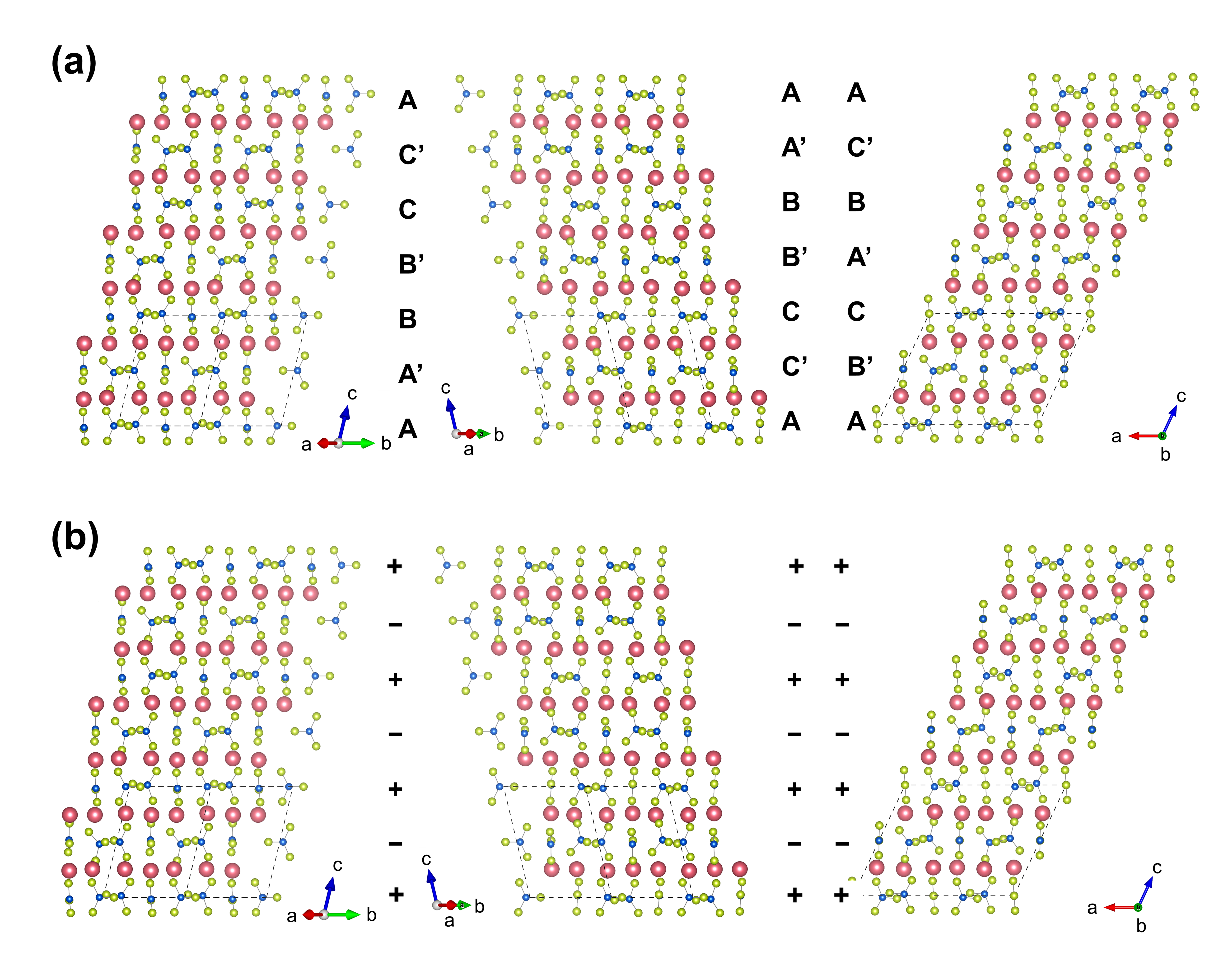


**Supplementary Fig. 7. The projections of vaterite structure with *C2* space group along the [0**$\bar{\boldsymbol{1}}$**0], [**$\bar{\boldsymbol{1}}\bar{\boldsymbol{1}}$**0] and [**$\boldsymbol{1}\bar{\boldsymbol{1}}$**0] directions.** The stacking order of carbonates was labelled according to different marking methods, while the method used in (a) comes from Demichelis’ work^22^ and (b) comes from Christy’s work^23^.


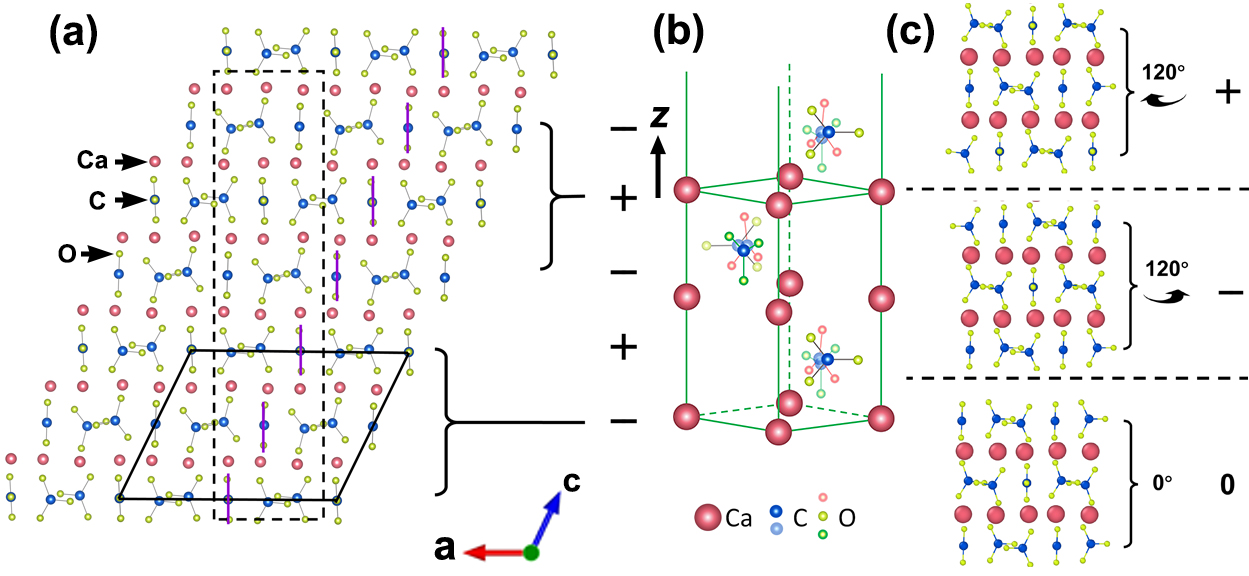


**Supplementary Fig. 8**. **Stacking features of vaterite.** (a) Structural projection of the monoclinic *C2* phase along the [010] direction. The shot purple lines indicate the stacking features of carbonate layers. The unit cell is denoted by the solid black lines, while the dashed box encloses a hexagonal lattice description of the *C2* structure. (b) Stereo schematic showing the general arrangements of calcium atoms and carbonates in vaterite. One of the C-O bonds should point towards the edge of the trigonal prism, resulting in three possible orientations of carbonates that occupy half of the Ca_6_ trigonal prismatic interstices. (c) The possible stacking sequences composed of three adjacent layers, which can be marked as “+”, “−”, and “0”, respectively.

**
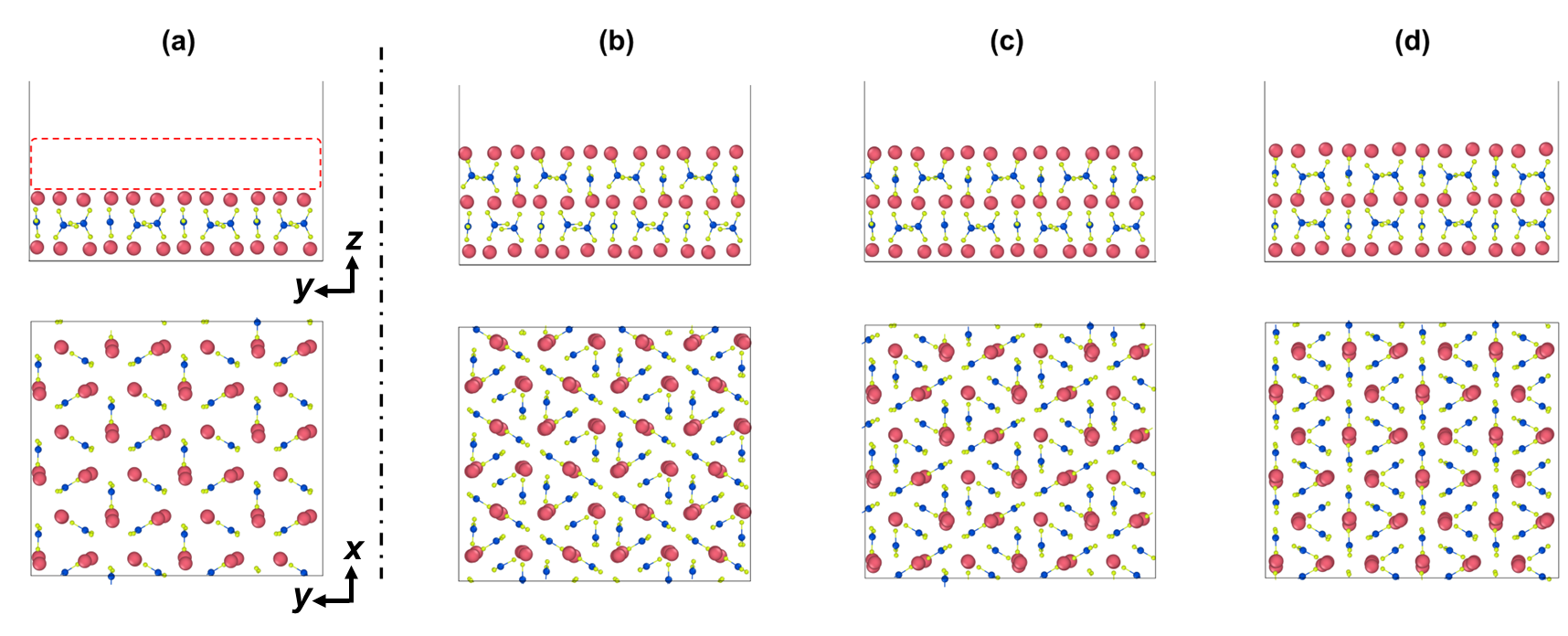
**

**Supplementary Fig. 9.** **Structural projections of only one-layer carbonates in the box on different planes.** (a) *yz-*plane (top) and *xy*-plane (bottom). (b-d) The projections of three possible structures after placing the second layer of carbonates on the next layer along the *z*-direction on the *yz*-plane (top) and *xy*-plane (bottom). The pink, blue, and yellow‒green spheres represent calcium atoms, carbon atoms and oxygen atoms, respectively.


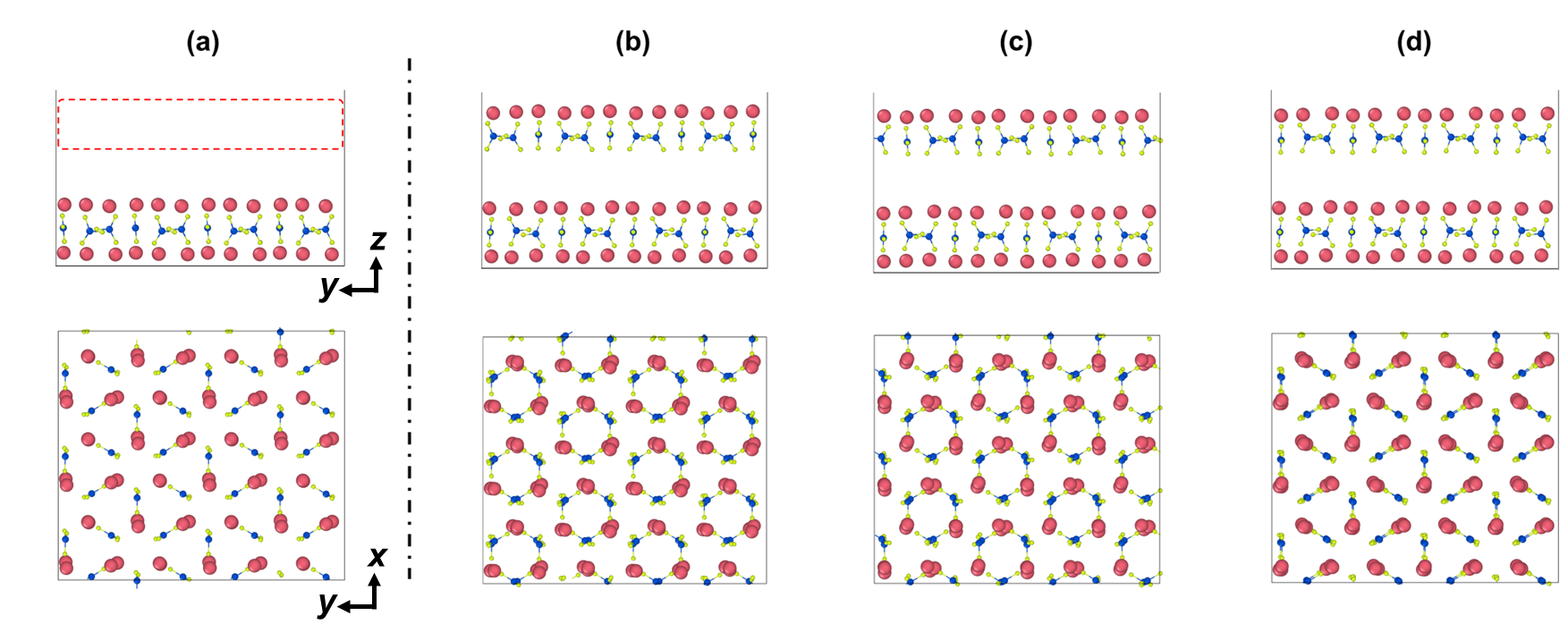


**Supplementary Fig. 10. Structural projections of only one-layer carbonates in the box on different planes.** (a) *yz*-plane (top) and *xy*-plane (bottom). (b-d) The projections of three possible structures after placing another layer of carbonates on the third layer of carbonates along the *z*-direction on the *yz*-plane (top) and *xy*-plane (bottom). The pink, blue, and yellow‒green spheres represent calcium atoms, carbon atoms and oxygen atoms, respectively.


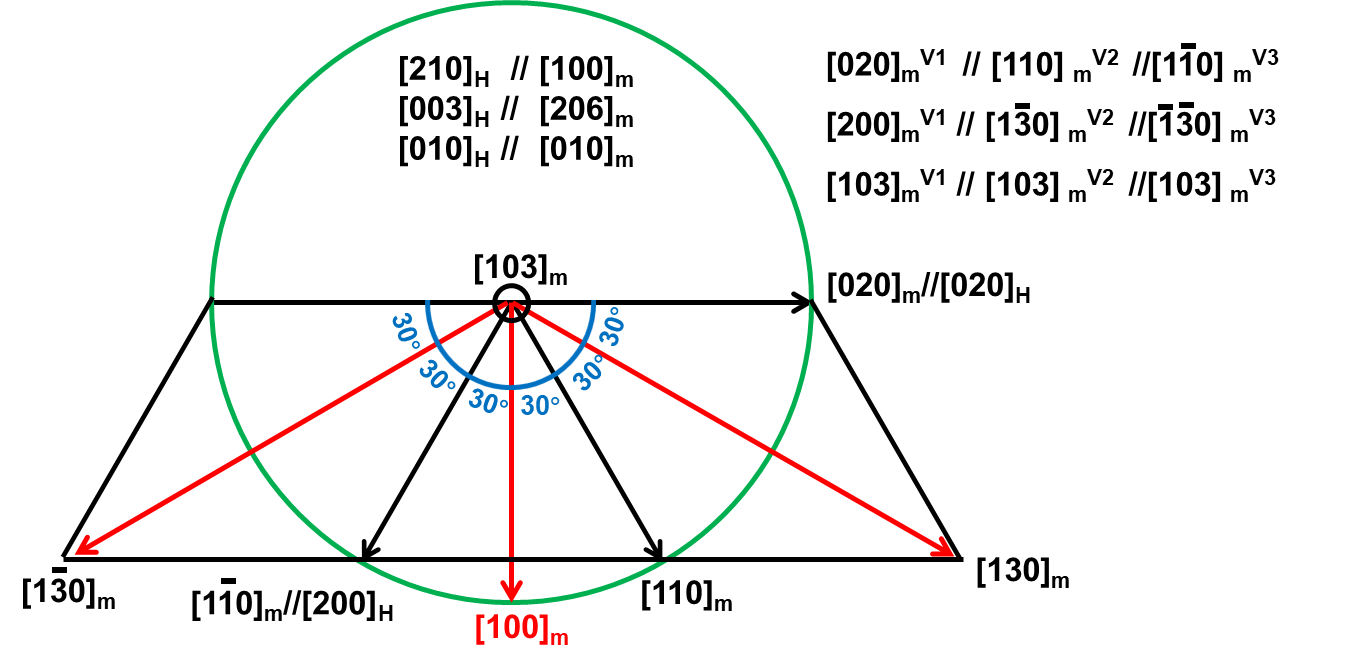


**Supplementary Fig. 11. Crystal orientations in monoclinic and hexagonal lattice.** Schematic showing the orientation relationship between the disordered Meyer structure with the space group of *P*6_3_/*mmc* and the monoclinic structure with the space group of *C2/c* or *Cc* or *C2*. The subscripts m and H indicate monoclinic latter and hexagonal lattices, respectively. The superscripts V1, V2 and V3 indicate three possible orientational variants within the monoclinic lattice.


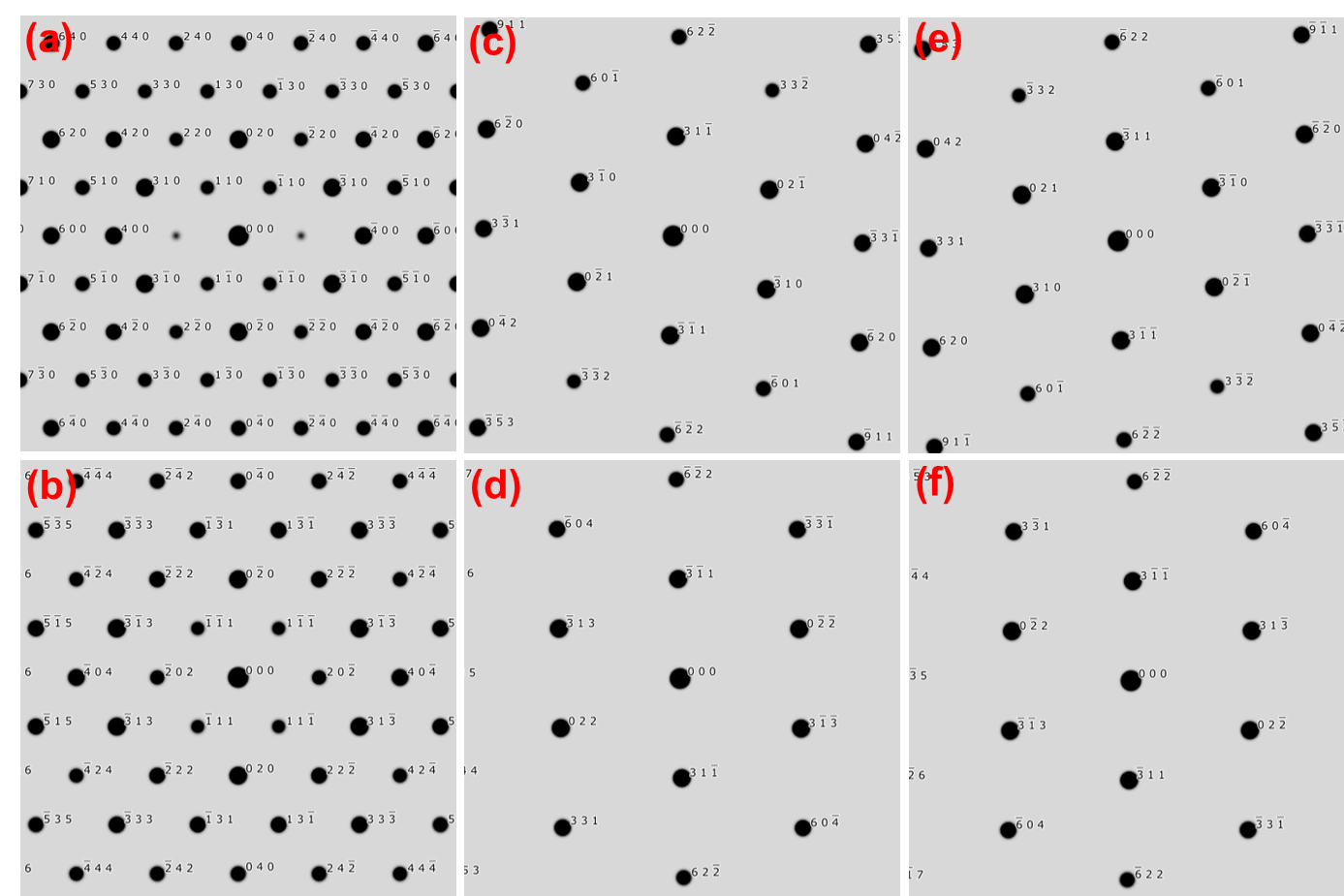


**Supplementary Fig. 12. Simulations of diffractions for *C2* variants with different orientations.** Simulated EDPs of (a, b) the ordered *C2*-V1 along the [001]_m_^V1^, [101] _m_^V1^ directions, (c, d) the ordered *C2*-V2 along the [136] _m_^V2^, [2$\bar{3}$3] _m_^V2^ directions, and (e, f) the ordered *C2*-V3 along the [1$\bar{3}$6] _m_^V3^, [233] _m_^V3^ directions.


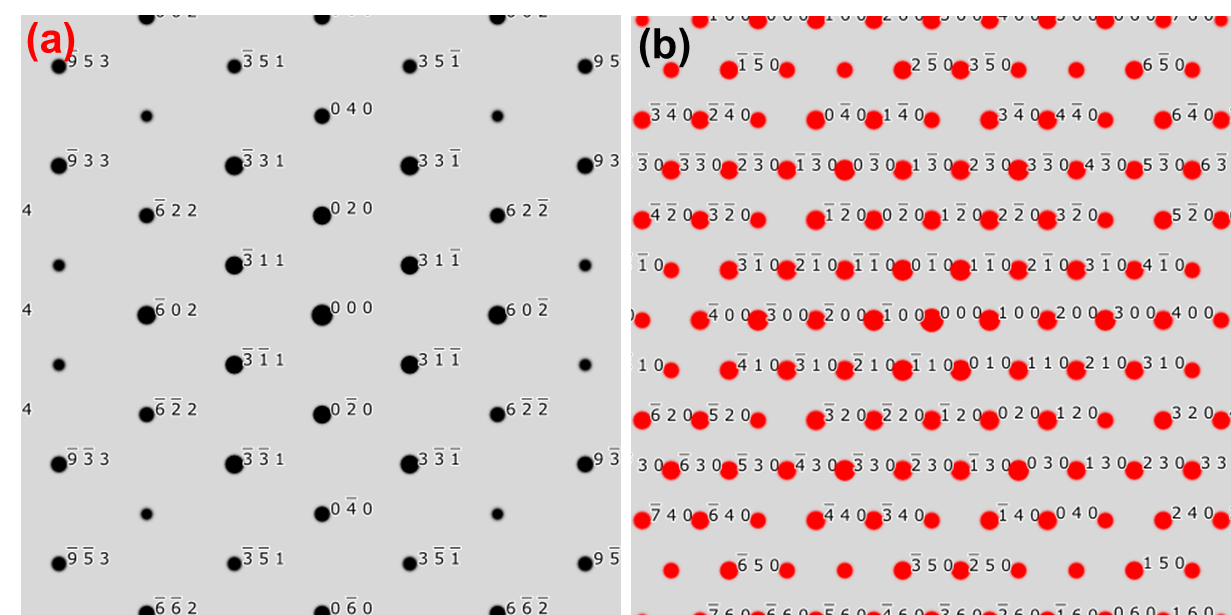


**Supplementary Fig. 13. Simulations of diffractions in the hexagonal and monoclinic lattice along the 6-fold or pseudo-6-fold axis.** Simulated EDPs of (a) the ordered *C2* structure along the [103]_m_ direction and (b) the disordered hexagonal Meyer structure along the [001]_H_ direction.


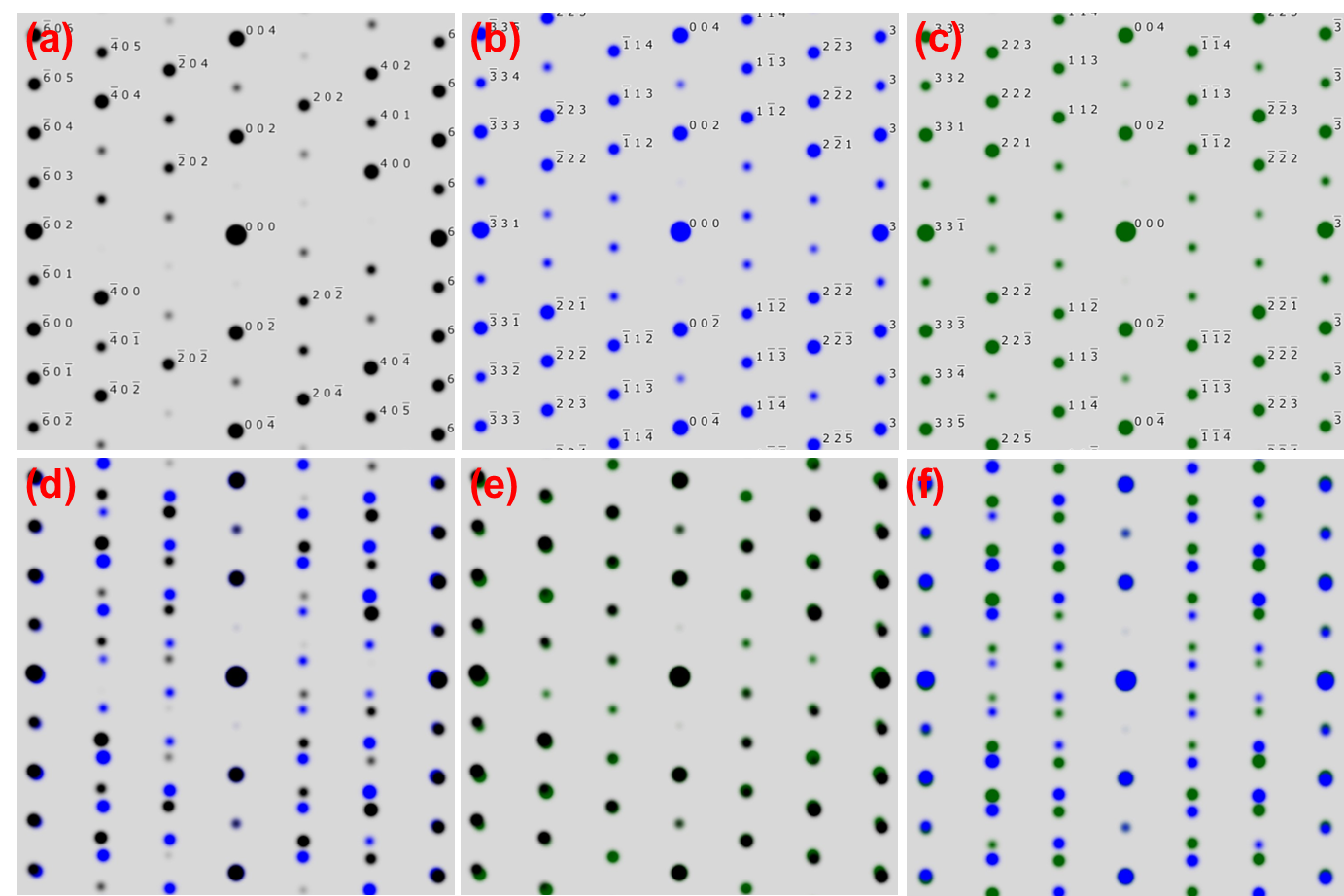


**Supplementary Fig. 14. Simulations of diffractions in *C2* structure with different variants.** Simulated EDPs of the ordered *C2* vaterite along (a) [010] _m_^V1^, (b) [110] _m_^V2^, (c) [1$\bar{1}0\text{]}\text{m}\text{V3}$ and composite EDPs of (d) [010] _m_^V1^ // [110] _m_^V2^, (e) [010] _m_^V1^ // [1$\bar{1}0\text{]}\text{m}\text{V3}$, (f) [110] _m_^V2^// [1$\bar{1}0\text{]}\text{m}\text{V3}$direction.


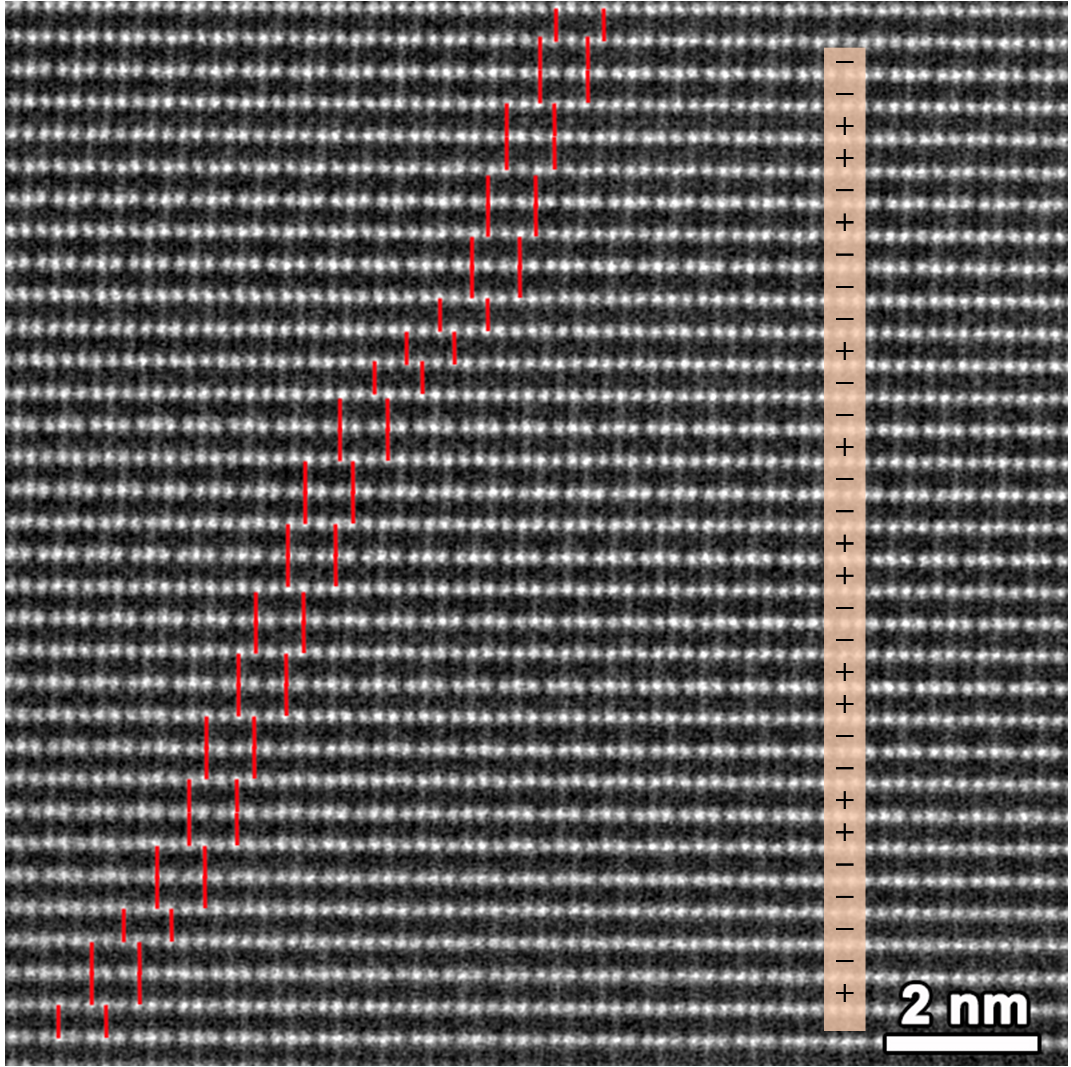


**Supplementary Fig. 15. Atomic resolution HAADF image with a large field of view along the [010]_m_ direction.** The image shows the polytypic features within vaterite along the [001]_m_ direction. The red vertical lines indicate the stacking feature of the Ca-C-O chains along the [001]_m_ direction. The inserted symbols indicate the stacking sequences of the carbonate layers introduced in Supplementary Fig. 8.


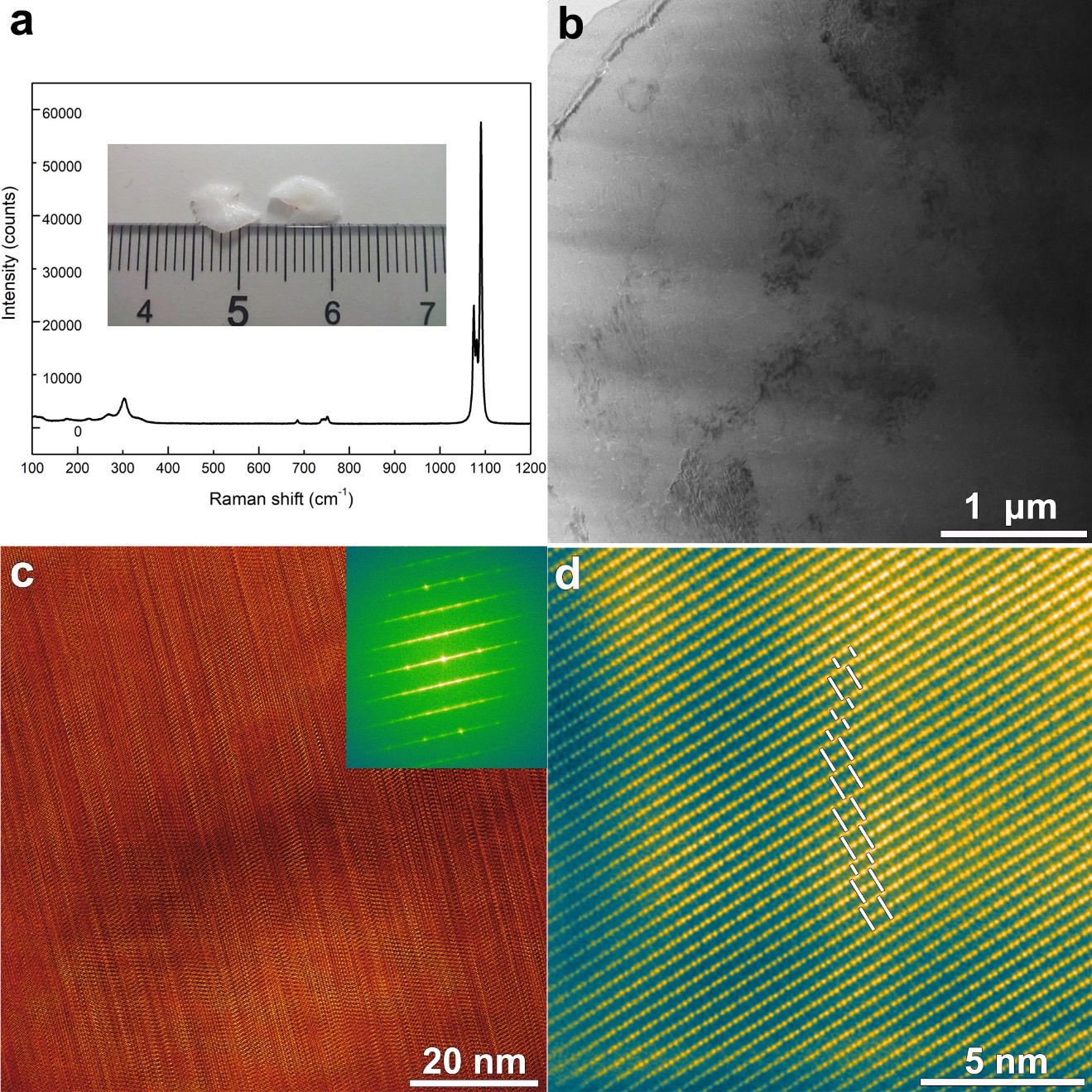


**Supplementary Fig. 16. Microstructural features for the asteriscus pairs of carp having vaterite structure.** (a) Raman spectrum. Inset is the optical image. (b) Low magnification TEM image. (c) HRTEM image and inset corresponding FFT patterns along [010]_m_ zone axis again showing the representative faulted feature in vaterite. (d) Atomic resolution HAADF image along the [010]_m_ zone axis showing the polytypic features of vaterite. The lines indicate the stacking feature of the Ca-C-O chains along the [001]_m_ direction.


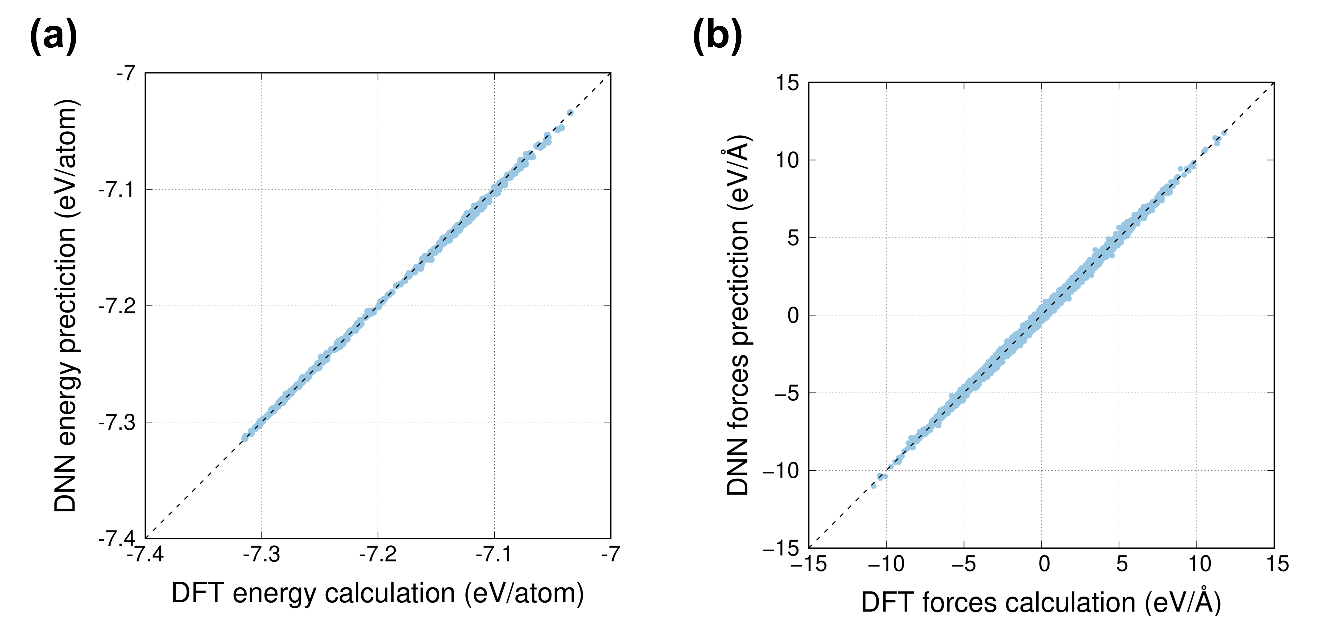


**Supplementary Fig. 17.** **Comparisons of the test set between the DFT calculation and deep neural network (DNN) model prediction.** (a) atomic energy. (b) forces. The configurations in the test set were extracted from MD trajectories using the DNN model under corresponding thermodynamic conditions.


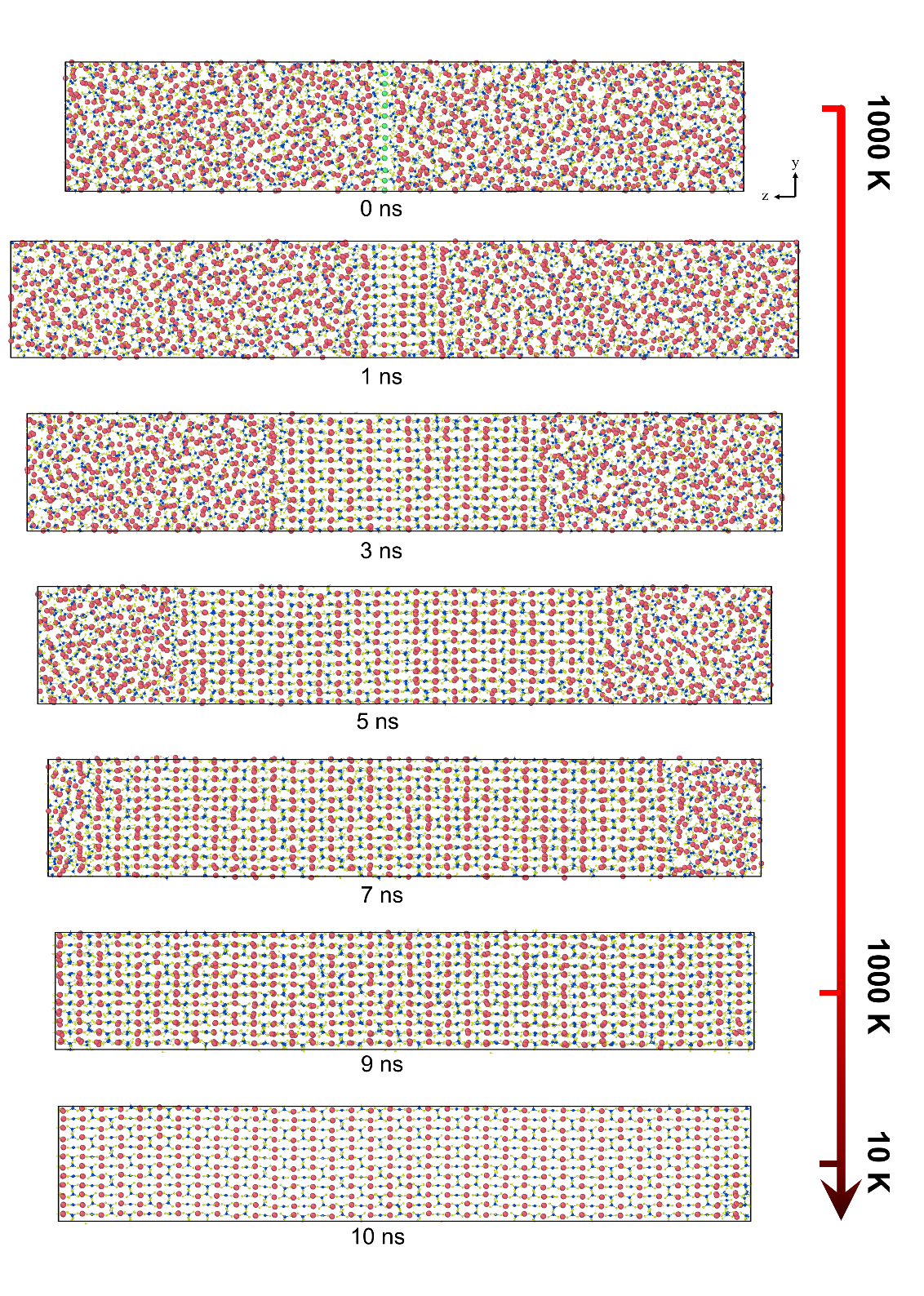


**Supplementary Fig. 18. Crystallization simulation of vaterite growth from liquid.** Snapshots of molecular dynamics simulations of vaterite growing along the *z*-axis direction during the first 9 *ns* at 1000 *K* after fixing a layer of calcium atoms and quenching to 10 *K* during the last 1 *ns*. The simulation cell contains 6480 atoms (1296 CaCO_3_ units). The green atoms in the snapshot of 0 *ns* indicate the fixed layer of calcium atoms. The pink, blue, and yellow‒green spheres represent calcium, carbon, and oxygen atoms, respectively.


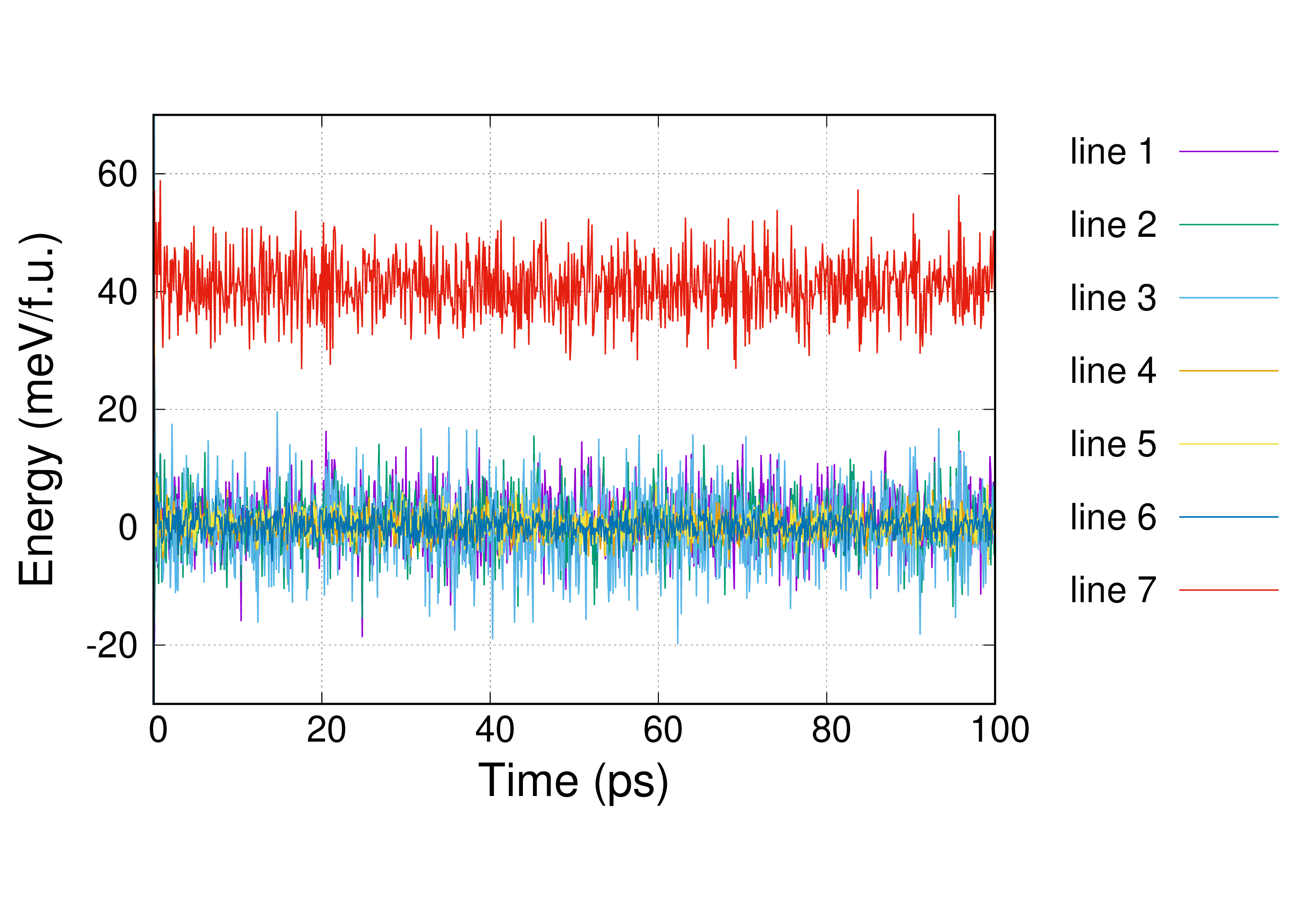


**Supplementary Fig. 19.** **The energy at 300 *K* for structures with different carbonate orientations as a function of time.** The line 7 represents the structure with the stacking sequence of “000000” (1080 atoms), while others represent the structures with the stacking sequence containing only “+” and/or “−”. (line 1: “++++++” (1080 atoms), line 2: “+−+−+−” (1080atoms), line 3: “++−−” (720 atoms), line 4, line 5 and line 6 represent the 36-layers structures (6480 atoms) obtained by MD simulations with disordered stacking sequences).


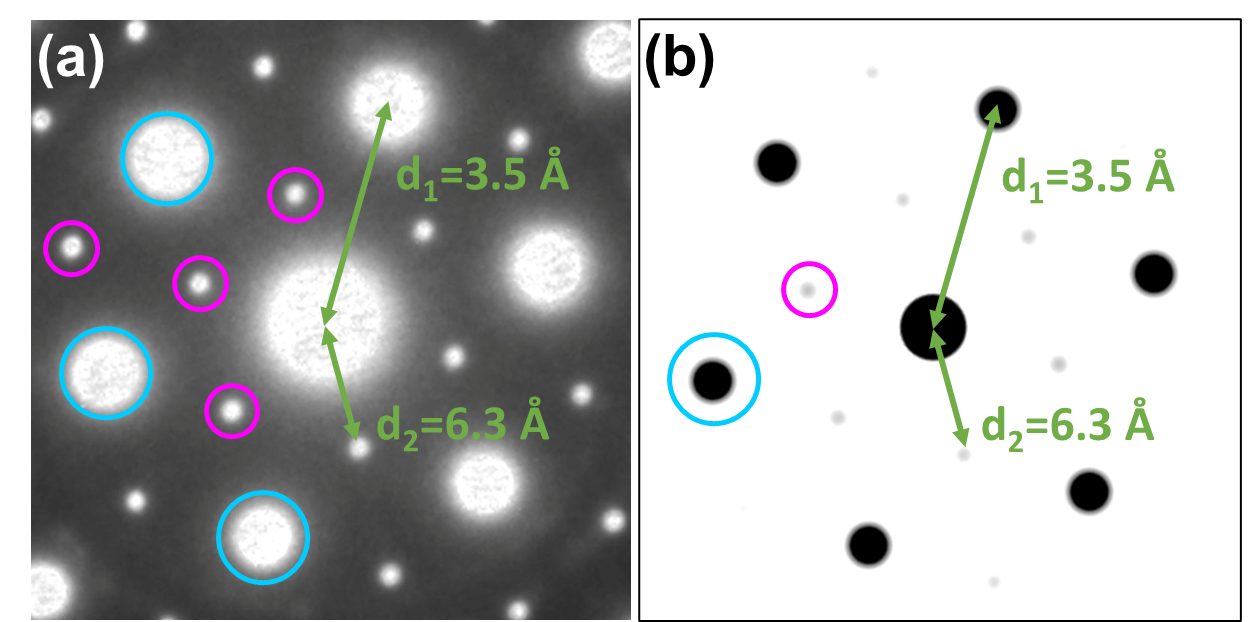


**Supplementary Fig. 20. Comparisons between experimental diffraction patterns and simulated ones.** (a) Local magnification of Fig. 2a EDPs. (b) Simulated EDPs of the theoretically grown polytypic structure along the stacking direction.


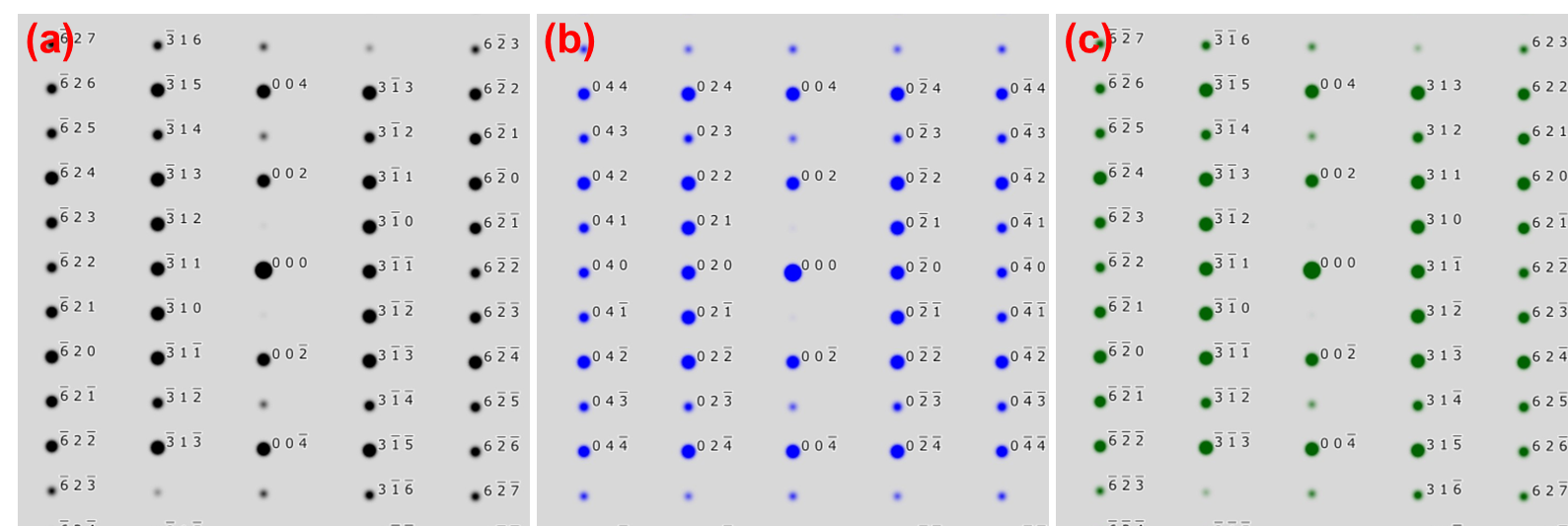


**Supplementary Fig. 21. Simulations of diffractions for *C2* structure with different variants.** Simulated EDPs of the ordered *C2* vaterite along the (a) [130]_m_^V1^, (b) [100] _m_^V2^ and (c) [1$\bar{3}0\text{]}\text{m}\text{V3}$ directions.


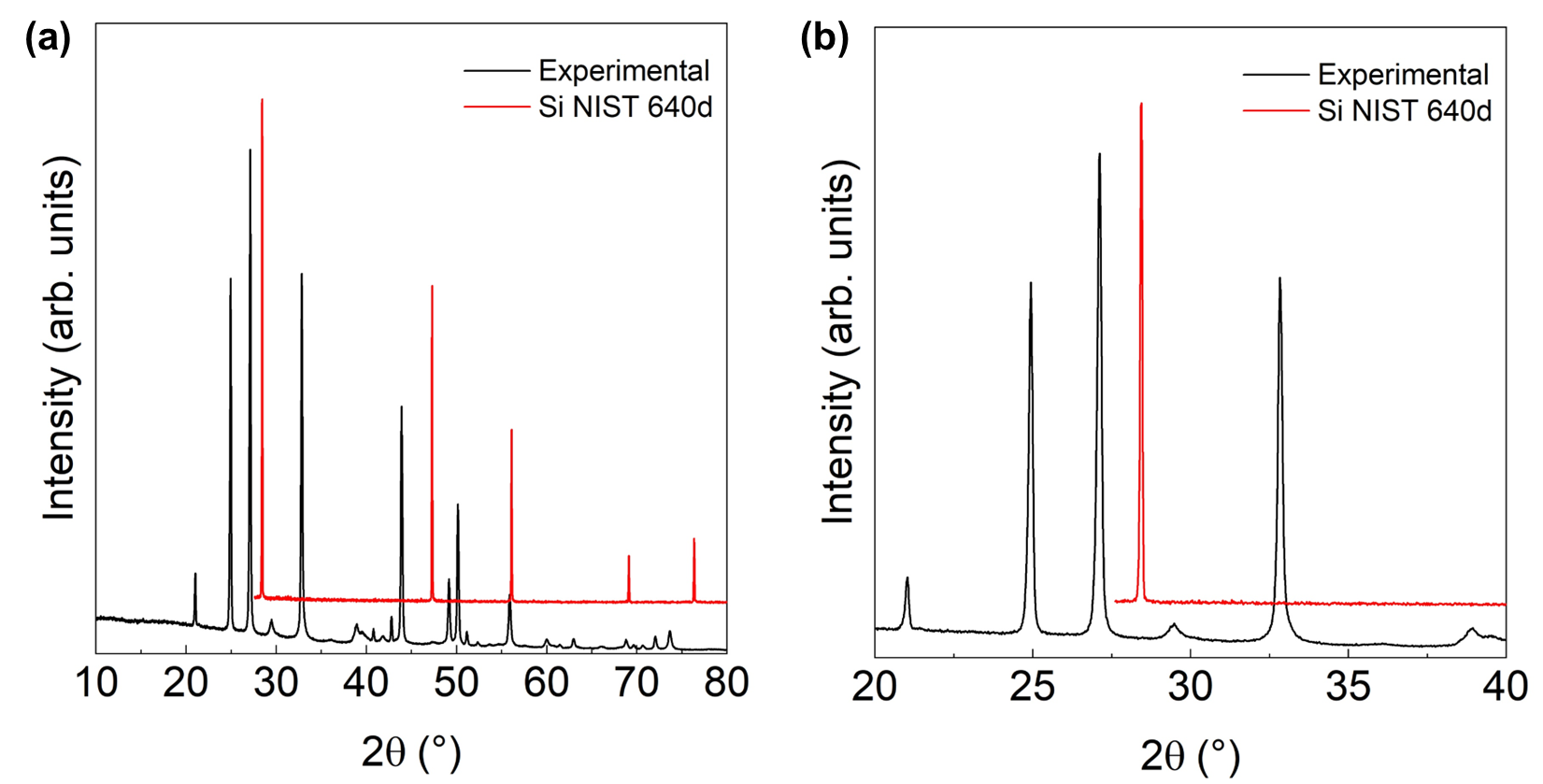


**Supplementary Fig. 22. Comparison of the experimental powder diffraction data against a Silicon standard (NIST 640d).** (a) XRD with a large field of view. (b) Zoomed in region of left figure. The reflections from Si are sharper than those from the sample indicating that the instrumental resolution is sufficient for probing broadening due to defects in the sample.


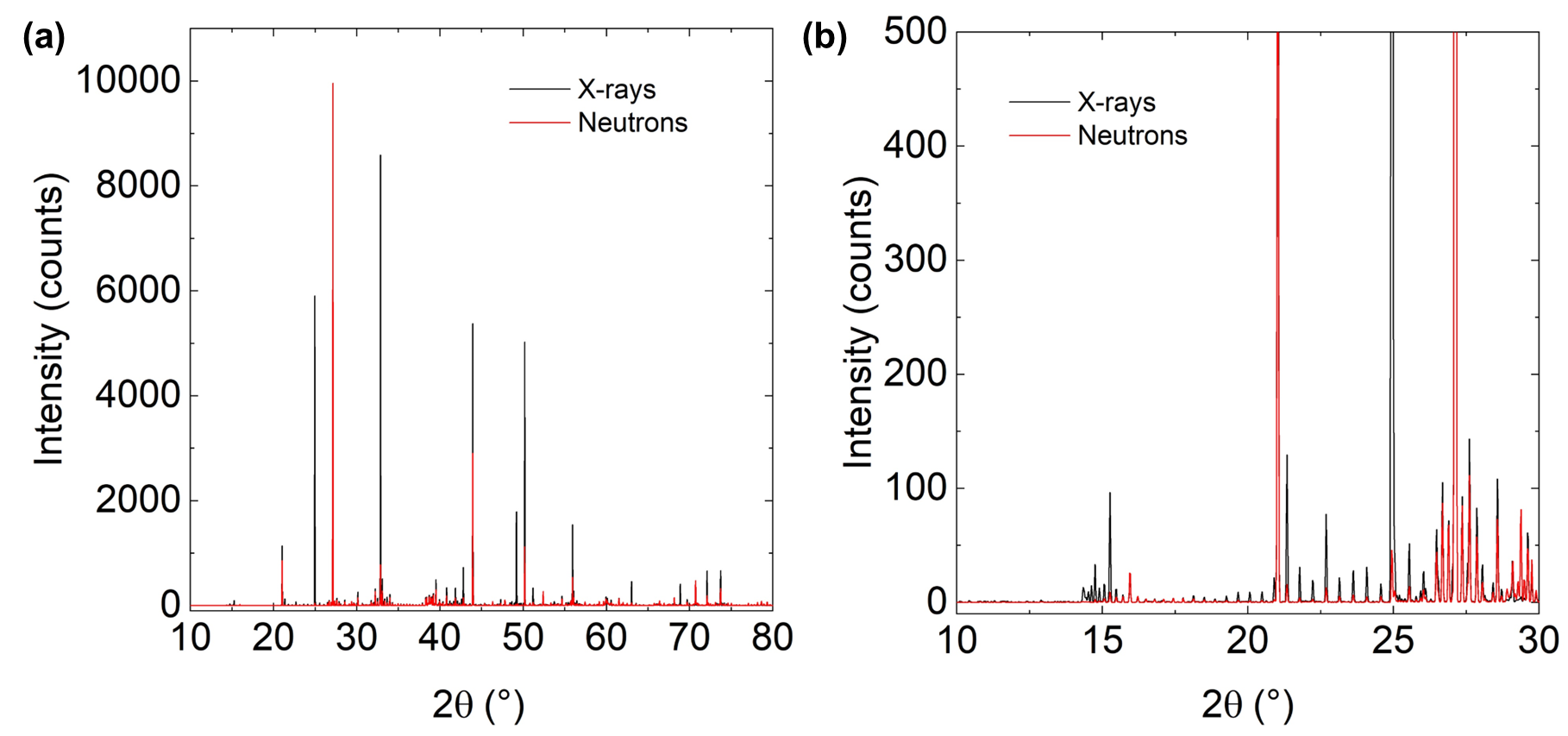


**Supplementary Fig. 23. Comparison of the simulated powder diffraction of the proposed model using X-ray and neutron of the same wavelength.** (a) Diffraction data with a large field of view. (b) Zoomed in region of left figure. Neither X-rays nor neutrons appear to give significant contrast in the entire *q*-range.


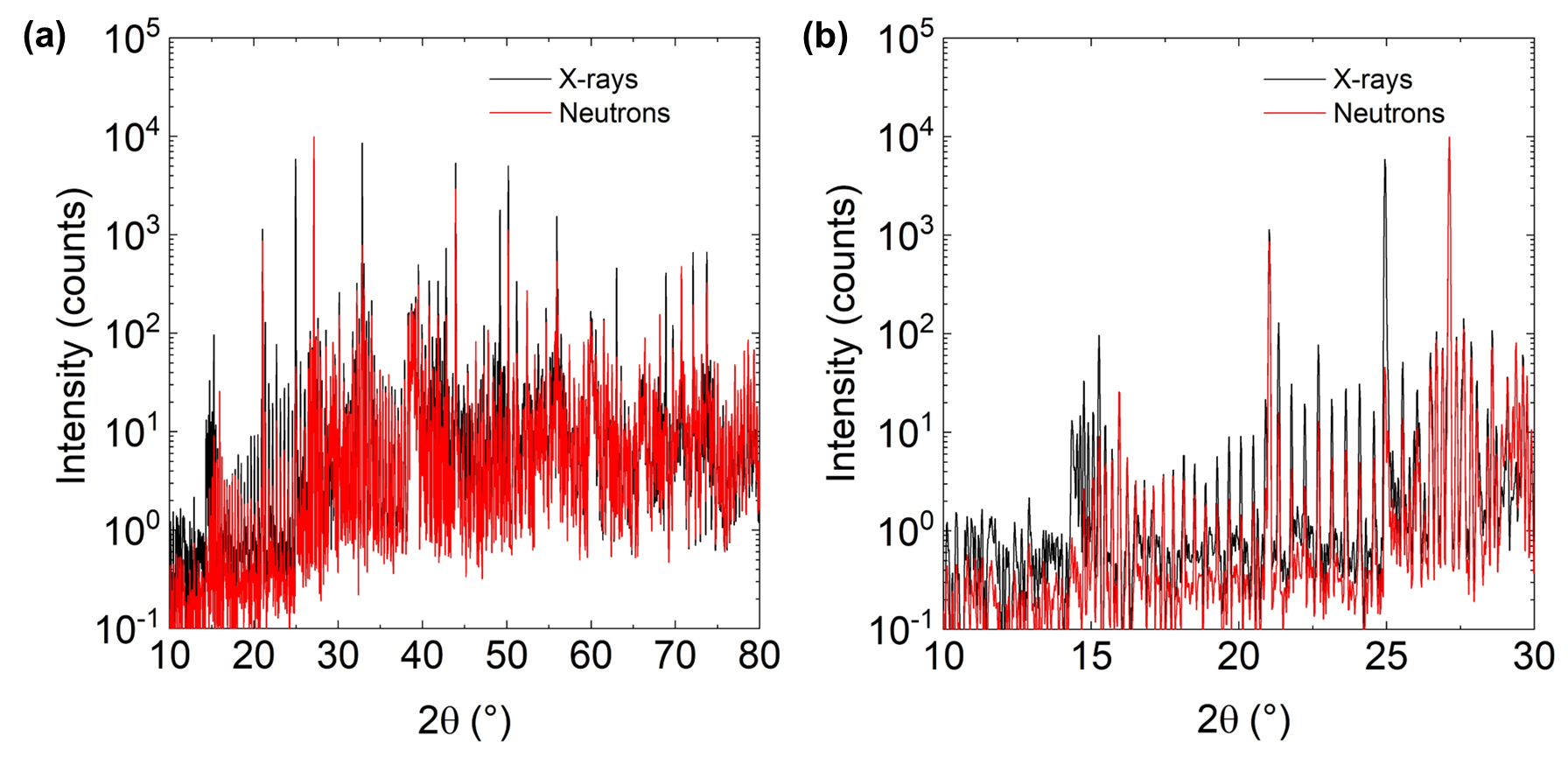


**Supplementary Fig. 24. Comparison of the simulated powder diffraction of the proposed model using X-ray and neutron of the same wavelength in logarithmic scale.** (a) A large field of view with Supplementary Fig. 23 in logarithmic scale. (b) Zoomed in region of left figure.


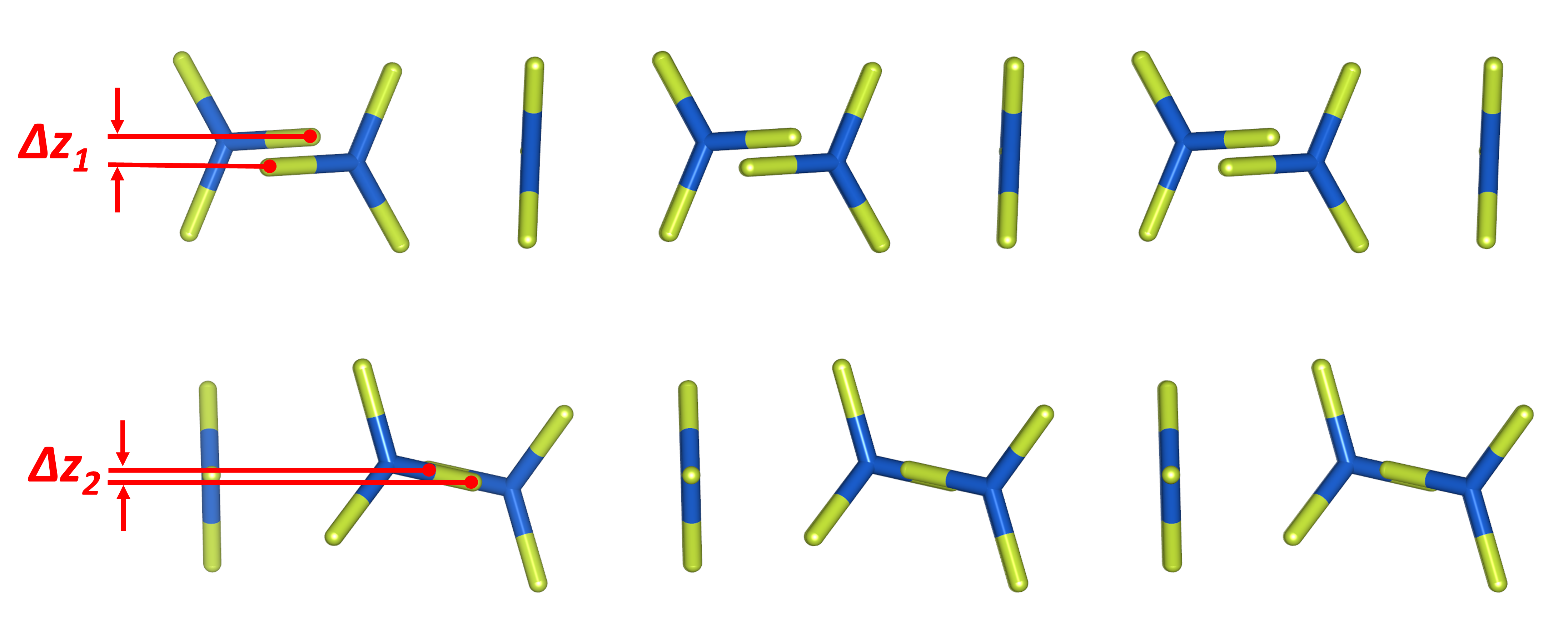


**Supplementary Fig. 25**. **Schematic diagram showing how collective variable are defined.** The carbonates are fragments in the *C2* structure with low symmetry.

**Supplementary References**

1 Gibson, R. E., Wyckoff, R. W. G. & Merwin, H. E. Vaterite and μ-calcium carbonate. *Am. J. Sci.* **s5-10**, 325-333 (1925).

2 Olshausen, S. Vaterite hexagonal cell determination by X-ray powder pattern. *Z. Kristallogr.* **61**, 463-464 (1925).

3 Meyer, H. J. Uber vaterit und seine struktur. *Angew. Chem., Int. Ed.* **71**, 678-678 (1959).

4 Kamhi, S. On the structure of vaterite CaCO_3_. *Acta Cryst.* **16**, 770-772 (1963).

5 Meyer, H. J. Struktur und fehlordnung des vaterits. *Z. Kristallogr.* **128** 183-212 (1969).

6 Le Bail, A., Ouhenia, S. & Chateigner, D. Microtwinning hypothesis for a more ordered vaterite model. *Powder Diffr.* **26**, 16-21 (2012).

7 Demichelis, R., Raiteri, P., Gale, J. D. & Dovesi, R. A new structural model for disorder in vaterite from first-principles calculations. *CrystEngComm* **14**, 44-47 (2012).

8 Mugnaioli, E. *et al.* *Ab initio* structure determination of vaterite by automated electron diffraction. *Angew. Chem. Int. Ed.* **51**, 7041-7045 (2012).

9 Wang, J. & Becker, U. Structure and carbonate orientation of vaterite (CaCO_3_). *Am. Mineral.* **94**, 380 (2009).

10 Kabalah-Amitai, L. *et al.* Vaterite crystals contain two interspersed crystal structures. *Science* **340**, 454-457 (2013).

11 Steciuk, G., Palatinus, L., Rohlicek, J., Ouhenia, S. & Chateigner, D. Stacking sequence variations in vaterite resolved by precession electron diffraction tomography using a unified superspace model. *Sci. Rep.* **9**, 9156 (2019).

12 Burgess, K. M. & Bryce, D. L. On the crystal structure of the vaterite polymorph of CaCO_3_: a calcium-43 solid-state NMR and computational assessment. *Solid State Nucl. Magn. Reson.* **65**, 75-83 (2015).

13 Wehrmeister, U., Soldati, A. L., Jacob, D. E., Häger, T. & Hofmeister, W. Raman spectroscopy of synthetic, geological and biological vaterite: a Raman spectroscopic study. *Journal of Raman Spectroscopy* **41**, 193-201 (2010).

14 Gabrielli, C., Jaouhari, R., Joiret, S. & Maurin, G. *In situ* Raman spectroscopy applied to electrochemical scaling. Determination of the structure of vaterite. *J. Raman Spectrosc.* **31**, 497-501 (2000).

15 Bryce, D. L., Bultz, E. B. & Aebi, D. Calcium-43 chemical shift tensors as probes of calcium binding environments. insight into the structure of the vaterite CaCO_3_ polymorph by 43Ca solid-state NMR spectroscopy. *J. Am. Chem. Soc.* **130**, 9282-9292 (2008).

16 Qiao, L. & Feng, Q. L. Study on twin stacking faults in vaterite tablets of freshwater lacklustre pearls. *J. Cryst. Growth* **304**, 253-256 (2007).

17 Medeiros, S. K., Albuquerque, E. L., Maia, F. F., Caetano, E. W. S. & Freire, V. N. First-principles calculations of structural, electronic, and optical absorption properties of CaCO_3_ Vaterite. *Chem. Phys. Lett.* **435**, 59-64 (2007).

18 McConnell, J. D. C. Vaterite from Ballycraigy, Larne, Northern Ireland. *Miner. Mag. J. Miner. Soc.* **32**, 535-544 (1960).

19 Dupont, L., Portemer, F. & late Michel Figlarz, t. Synthesis and study of a well crystallized CaCO_3_ vaterite showing a new habitus. *J. Mater. Chem.* **7**, 797-800 (1997).

20 Chakoumakos, B. C., Pracheil, B. M., Koenigs, R. P., Bruch, R. M. & Feygenson, M. Empirically testing vaterite structural models using neutron diffraction and thermal analysis. *Sci. Rep.* **6**, 36799 (2016).

21 Wang, J. *et al.* Carbonate orientational order and superlattice structure in vaterite. *J. Cryst. Growth* **407**, 78-86 (2014).

22 Demichelis, R., Raiteri, P., Gale, J. D. & Dovesi, R. The multiple structures of vaterite. *Cryst. Growth. Des.* **13**, 2247-2251 (2013).

23 Christy, A. G. A review of the structures of vaterite: the impossible, the possible, and the likely. *Cryst. Growth. Des.* **17**, 3567-3578 (2017).
